# Multi-Omics Analysis of Lipid Metabolism for a Marine Probiotic *Meyerozyma guilliermondii* GXDK6 Under High NaCl Stress

**DOI:** 10.3389/fgene.2021.798535

**Published:** 2022-01-13

**Authors:** Huijie Sun, Xinghua Cai, Bing Yan, Huashan Bai, Duotao Meng, Xueyan Mo, Sheng He, Guijiao Su, Chengjian Jiang

**Affiliations:** ^1^ State Key Laboratory for Conservation and Utilization of Subtropical Agro-bioresources, Guangxi Research Center for Microbial and Enzyme Engineering Technology, College of Life Science and Technology, Guangxi University, Nanning, China; ^2^ Guangxi Key Lab of Mangrove Conservation and Utilization, Guangxi Mangrove Research Center, Guangxi Academy of Sciences, Beihai, China; ^3^ Guangxi Birth Defects Prevention and Control Institute, Maternal and Child Health Hospital of Guangxi Zhuang Autonomous Region, Nanning, China.

**Keywords:** *Meyerozyma guilliermondii*, lipid metabolism, integrated omics technology, signal transduction, antibiotics

## Abstract

Investigating microbial lipid regulation contributes to understanding the lipid-dependent signal transduction process of cells and helps to improve the sensitivity of microorganisms to environmental factors by interfering with lipid metabolism, thus beneficial for constructing advanced cell factories of novel molecular drugs. Integrated omics technology was used to systematically reveal the lipid metabolism mechanism of a marine *Meyerozyma guilliermondii* GXDK6 under high NaCl stress and test the sensitivity of GXDK6 to antibiotics when its lipid metabolism transformed. The omics data showed that when GXDK6 perceived 10% NaCl stress, the expression of *AYR1* and NADPH-dependent 1-acyldihydroxyacetone phosphate reductase was inhibited, which weaken the budding and proliferation of cell membranes. This finding was further validated by decreased 64.39% of OD_600_ under 10% NaCl stress when compared with salt-free stress. In addition, salt stress promoted a large intracellular accumulation of glycerol, which was also verified by exogenous addition of glycerol. Moreover, NaCl stress remarkably inhibited the expression of drug target proteins (such as lanosterol 14-alpha demethylase), thereby increasing sensitivity to fluconazole. This study provided new insights into the molecular mechanism involved in the regulation of lipid metabolism in *Meyerozyma guilliermondii* strain and contributed to developing new methods to improve the effectiveness of killing fungi with lower antibiotics.

## Introduction

Microbial lipid metabolism plays a key role in cell signaling, osmotic pressure balance, and the construction of cell membrane structures (Wang S. et al., 2021; Klug and Daum, 2014). Interfering with the lipid metabolism of microorganisms could significantly change their sensitivity to the environment and transform their life activities, thereby contributing to the targeted development of new microbial cell factories (Ferreira et al., 2018). The lipid metabolism in the Kyoto Encyclopedia of Genes and Genomes (KEGG) database is currently known to have 17 branch pathways. Among them, steroid biosynthesis and sphingolipid metabolism are relevant to the microorganisms’ cytoskeleton, which regulates their membrane fluidity and permeability (Kraft, 2017; Xu and Li, 2020). Glycerolipid metabolism is closed related to the construction of cell membrane structure (phospholipids and other lipids, varying by organelle type) and energy storage (primarily in the form of triacylglycerol) (Zhang and Reue, 2017). Glycerophospholipid metabolism is mainly linked to cell growth and proliferation (Nebauer et al., 2004). In addition, fatty acid metabolism (such as fatty acid biosynthesis, fatty acid degradation, fatty acid elongation, and biosynthesis of unsaturated fatty acids) are closely related to microbial cell signal transduction, and they are widely considered to be responsible for participating in and responding to extracellular environmental signal factors (Ross et al., 2003; Fan et al., 2016). These pathways of lipid metabolism are known to be indispensable for the basic life activities of microorganisms by supporting microbes to make precise physiological responses in different environments. Therefore, the lipid metabolism of microorganisms is important to forming the cytoskeleton and responding to environmental signal factors, thus enabling microorganisms to exhibit unique life activities in different environments.

**GRAPHICAL ABSTRACT:**
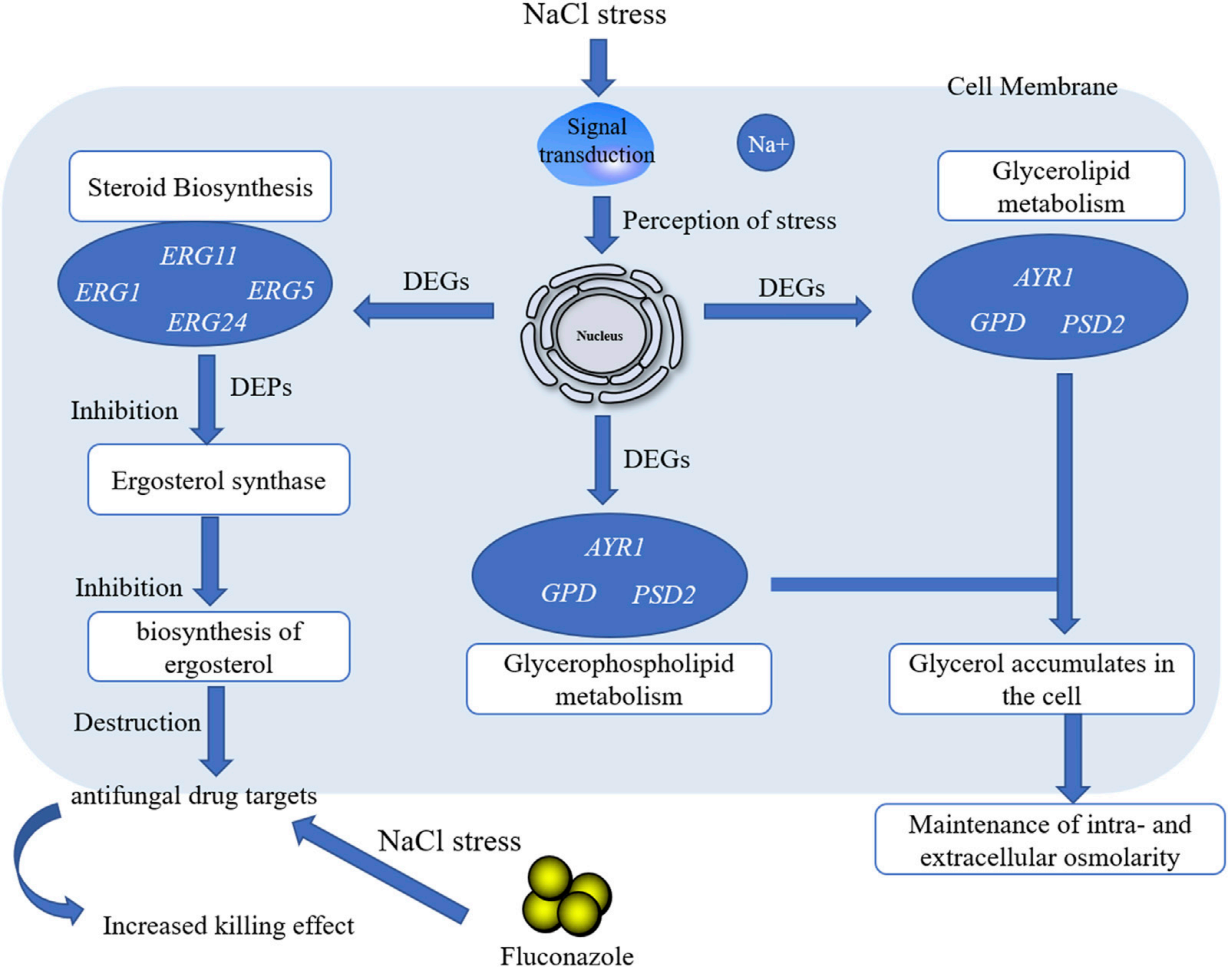


The microbial lipid metabolism mechanism has gained increasing interest. Guo *et al.* reported that when *Saccharomyces cerevisiae* was stressed by strong acid, its lipid products were remodeled, which was helpful for improving cell viability under acid stress (Guo et al., 2018). Randez-Gil *et al.* demonstrated that lipid metabolism was involved in regulating the composition of cell membranes in *S. cerevisiae* under low-temperature conditions (Randez-Gil et al., 2018); Zhou *et al.* engineered *S. cerevisiae* to produce L-alkenes by manipulating fatty acid metabolism, enzyme selection, and the electron transfer system and expressing a transporter (Zhou et al., 2018). Therefore, the regulation of lipid metabolism is a useful method for microorganisms to enhance their survival ability under different adverse environments. However, few reports on the mechanism of lipid metabolism focused on how the lipids work and/or how this mechanism contributed to cells (Casanovas et al., 2015; Wang et al., 2018; Lu et al., 2021). A global regulatory network of lipid metabolism has not been revealed yet, resulting in random transformation, which is not conducive to accurately constructing advanced cell factories (Galafassi et al., 2015; Qi et al., 2017; Tian et al., 2019; Li C. et al., 2021). Therefore, mapping the lipid regulation network of microorganisms is an important prerequisite for constructing advanced cell factories by interfering with lipid metabolism.

In the previous study, a marine *Meyerozyma guilliermondii* GXDK6 that contained abundant lipid metabolism genes was obtained from subtropical marine mangrove microorganisms, and it showed excellent salt-tolerant survivability (14% NaCl and 18% KCl) (Mo et al., 2021). On the basis of its typical physicochemical characteristics, GXDK6 was hypothesized to have a unique regulatory network of lipid metabolism. GXDK6 can precisely regulate the expression of genes and/or proteins related to lipid metabolism to support its survival under salt stress conditions, which lead to greater sensitivity to certain environmental factors. The lipid regulation mechanism of GXDK6 under high-salt stress was systematically investigated by using an integrative omics technology (whole-genomic, transcriptomic, and proteomic) to test these hypotheses. The systematic analysis of the regulation network of lipid metabolism in *M. guilliermondii* strain provide a feasible reference for the construction of lipid cell factories.

## Materials and Methods

### Experimental Strain

A yeast *M. guilliermondii* GXDK6 was obtained from the subtropical marine mangrove sediments, and was released by China General Microbiological Culture Collection Center (CGMCC) with the preservation number CGMCC No. 16007.

### Effect of NaCl Stress on the Survival of *M. guilliermondii* Strain

GXDK6 was cultured at different NaCl concentrations (0, 5, and 10%) with 37°C and 200 rpm for 0–48 h, respectively. Its growth curve was evaluated by turbidimetric method. The cells were collected by centrifugation at 12,000 rpm for 10 min and then washed three times with sterile saline (0, 5, and 10% NaCl). The GXDK6 was further prepared into cell suspension and observed using scanning electron microscopy (SEM, Thermoelectric Company, American) to explore the influence of NaCl stress on the morphology.

### Whole-Genome Analysis for Lipid Metabolism Genes in *M. guilliermondii* Strain

GXDK6 was incubated by using an enrichment technique and GBM liquid medium (0.2% yeast extract, 0.2% beef extract, 0.5% polypeptone, 0.6% sucrose, pH 7.0) with 200 rpm and 30°C for 8 h. The cells were then collected by centrifugation at 8,000 rpm and 4°C for 10 min and washed repeatedly with 0.1 mol/L PBS buffer. The purity of DNA in GXDK6 (extracted by CTAB method) was verified by PCR (Polymerase Chain Reaction) and agarose gel electrophoresis, and then the ITS gene was amplified by PCR (Mo et al., 2021). The ITS sequencing data of GXDK6 were deposited to the National Microbiology Data Center database (http://nmdc.cn) under the Accession Number of NMDCN000022O. The whole-genome sequencing and analysis of GXDK6 were performed by the BGI Genomics Co., Ltd (Shenzhen, China). An online software FastQC (http://www.bioinformatics.babraham.ac.uk/projects/fastqc) was used for the quality control of the second-generation sequencing downtime data. To determine the percentage of single-copy genes in the total single-copy genes, an online software BUSCO (http://busco.ezlab.org, v3.0.2) was conducted to complete the sequence comparison of the genome sequences. The genes related to lipid metabolism in GXDK6 were blasted and analyzed by whole genome method. The pathway enrichment analysis of genes regulating lipid metabolism was annotated according to the KEGG database.

### Transcriptome Analysis of *M. guilliermondii* Strain

GXDK6 was incubated for 16 h at 0, 5, and 10% NaCl, respectively. The cells were then collected by centrifugation at 12,000 rpm for 10 min. Total RNA was extracted using Trizol method (Villa-Rodríguez et al., 2018) (Trizol reagent kit, Invitrogen, Carlsbad, CA, United States). RNA quality was assessed on an Agilent 2,100 Bioanalyzer (Agilent Technologies, Palo Alto, CA, United States) and checked using rnase free agarose gel electrophoresis. After total RNA was extracted, eukaryotic mRNA was enriched by Oligo (dT) beads, while prokaryotic mRNA was enriched by removing rRNA by Ribo-ZeroTM Magnetic Kit (Epicentre, Madison, WI, United States). Then the enriched mRNA was fragmented into short fragments using fragmentation buffer and reverse transcripted into cDNA with random primers. Second-strand cDNA were synthesized by DNA polymerase I, rnase H, dNTP and buffer. Then the cDNA fragments were purified with QiaQuick PCR extraction kit (Qiagen, Venlo, Netherlands), end repaired, poly(A) added, and ligated to Illumina sequencing adapters. The ligation products were size selected by agarose gel electrophoresis, PCR amplified, and sequenced using Illumina HiSeq2500 by Gene Denovo Biotechnology Co. (Guangzhou, China). RNAs differential expression analysis was performed by DESeq2 (Love et al., 2014) software between two different groups (and by edgeR between two samples) (Robinson et al., 2010). The genes/transcripts with the parameter of false discovery rate (FDR) below 0.05 and absolute fold change≥2 were considered differentially expressed genes/transcripts. All samples for transcriptome sequencing were set up in three parallel.

### Proteomic Analysis of *M. guilliermondii* Strain

The incubation and cell collection of GXDK6 were consistent with the above method. The proteins in GXDK6 were extracted by SDT Lysis methods (Zhu et al., 2014) and separated on 12.5% SDS-PAGE gel. Protein bands were visualized by Coomassie Blue R-250 staining. The detergent, DTT and other low-molecular-weight components were removed using UA buffer by repeated ultrafiltration (Sartorius, 30 kD). Then 100 μL iodoacetamide (100 mM IAA in UA buffer) was added to block reduced cysteine residues. After washing, the protein suspensions were digested with 4 μg trypsin (Promega) in 40 μL 0.1 M TEAB buffer overnight at 37°C, and the resulting peptides were collected as a filtrate. The peptide content was estimated by UV light spectral density at 280 nm using an extinctions coefficient of 1.1 of 0.1% (g/L) solution. Sequencing of the extracted proteins was conducted by Gene Denovo Biotechnology Co. (Guangzhou, China) by using a tandem mass tag (TMT)-based quantitative proteomics (Myers et al., 2019). TMT labeled peptides were fractionated by RP chromatography using the Agilent 1,260 infinity II HPLC. LC-MS/MS analysis was performed on a Q Exactive plus mass spectrometer (Thermo Fisher Scientific) that was coupled to Easy nLC (Thermo Fisher Scientific) for 60/90 min. MS/MS raw files were processed using MASCOT engine (Matrix Science, London, United Kingdom; version 2.6) embedded into Proteome Discoverer 2.2, and searched against the NCBInr/UniProt database. Proteins with Fold change>1.2 and *p* value (Student’s *t* test) < 0.05 were considered as differentially expressed proteins.

### Fluconazole Resistance Test of *M. guilliermondii* Strain

GXDK6 was inoculated under 0, 5 and 10% NaCl stress containing fluconazole (64 μg/ml). The culture conditions under 0, 5 and 10% NaCl stress without fluconazole were set as the controls. Turbidity method and spread plate method was used to investigate the effect of salt stimulation on the drug resistance ability of GXDK6.

### RT-qPCR Verification of Transcriptome Data

According to transcriptome and whole-genome data, *GUT1* (encoding glycerol kinase), *ADH7* (encoding NADP-dependent alcohol dehydrogenase 7), *GPP1* (encoding glycerol-1-phosphate phosphohydrolase 1) were selected and designed (Primer Premier 5.0 software) for qPCR verification in this work (Supplementary Table S18) [the internal reference gene refers to Díaz et al. (2013)]. The reaction volume of RT-qPCR was 20 μL. The mixture was heated to 95°C for 2 min, followed by 40 cycles of denaturation at 95°C for 15 s, and annealing/extension at 60°C (depending on the primers) for 30 s. The cycling temperature was then increased by 0.3°C every 10 s from 63 to 95°C to obtain the melting curve. Three sets of parallel trials were set up for each gene. Real-time PCR was carried out using a high-throughput real-time fluorescence quantitative PCR instrument ROCHE 480 (ROCHE, United States). Meanwhile, fluorescence detection was performed on LightCycler^®^ 480 Software release 1.5.1.62 SP3, and the number of gene amplification cycles was counted in the Software. Relative quantitation was calculated using the 2^−ΔΔCt^ method (Livak and Schmittgen, 2001).

### Effect of Adding Exogenous Glycerol on the Growth of *M. guilliermondii* Strain

Exogenous addition of glycerol was used to verify the transcriptome and proteome results. The salt tolerance of GXDK6 was investigated by adding 120 mg/L, 1,200 mg/L and 12,000 mg/L of exogenous glycerol to salt-containing medium. The liquid medium without glycerol and containing 0, 5 and 10% NaCl was also set up as a control. Subsequently, OD_600_ was detected to evaluate the effect of adding exogenous glycerol on the growth of GXDK6.

### Data Analysis

Experimental data were processed with SPSS 25, Origin 2018, Excel 2019, TBtools, and Diamond software. The relevant query databases were Uniprot, SWISS-MODEL, KEGG, and NCBI. Significant difference was at a *p*-value < 0.05.

## Results and Discussion

### The Growth Status was Changed When *M. guilliermondii* Strain Perceived Salt Stress

**FIGURE 1:**
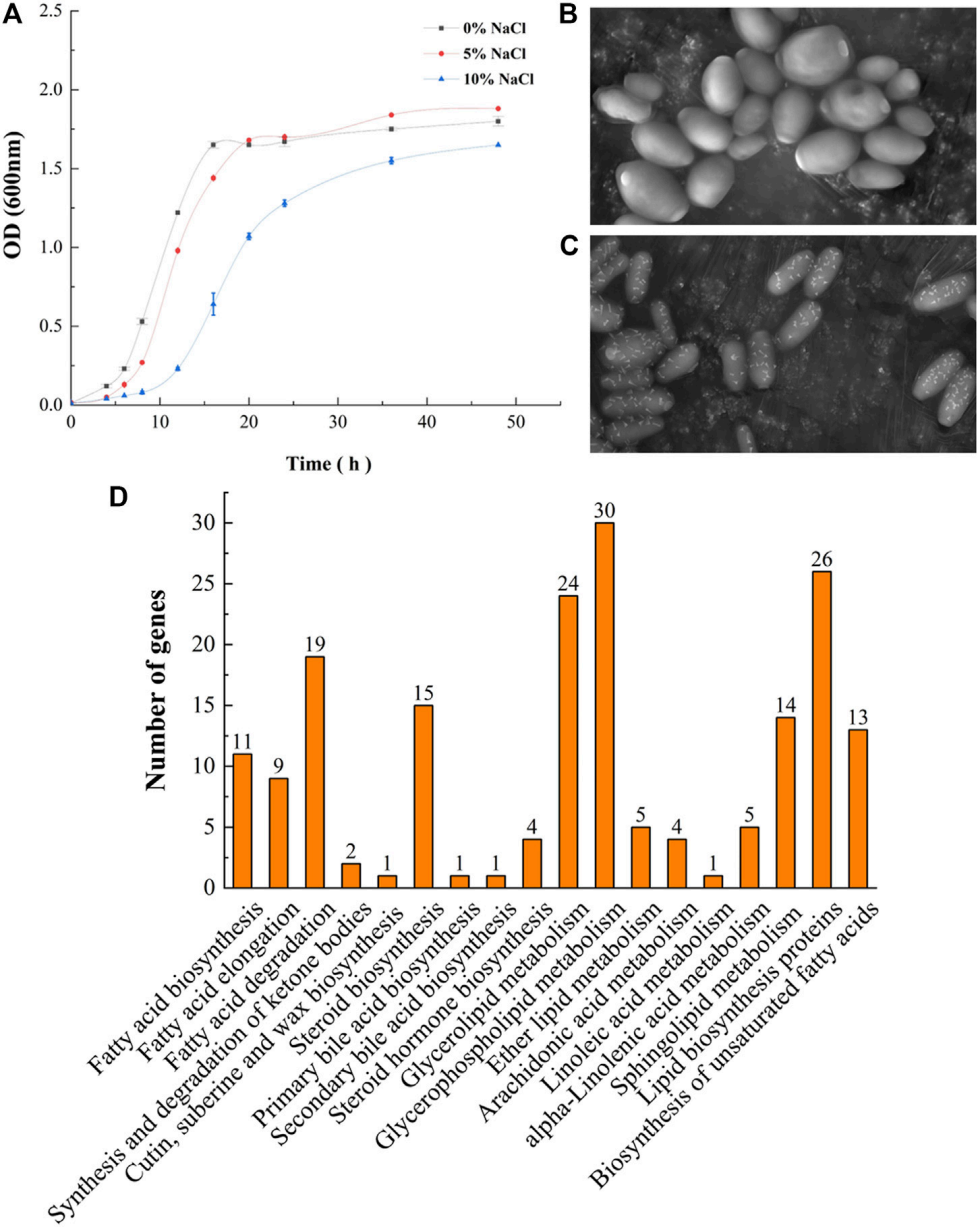
Growth of GXDK6 under NaCl stress. **(A)** Growth curve of GXDK6 under different NaCl stresses; **(B)** morphology of GXDK6 under 0% NaCl; **(C)** morphology of GXDK6 under 10% NaCl; **(D)** Gene enrichment analysis of lipid metabolism.

The lag phase of GXDK6 under 5–10% NaCl stress was prolonged to 4 h, which was approximately 2 h longer than that of the controls, indicating that the DNA replication and transcription of GXDK6 were inhibited by NaCl stress (Figure 1A). Besides, the growth of GXDK6 in log phase was remarkably inhibited by NaCl stress. With the incubation time at 16 h as an example, the OD_600_ of GXDK6 from non-NaCl stress condition was 1.567; it decreased to 1.281 (decreased by 18.25%) under 5% NaCl stress and 0.558 under 10% NaCl stress (decreased by 64.39%), suggesting that the metabolism and protein expression of GXDK6 were transformed by NaCl stress. However, the OD_600_ value of GXDK6 in stable phase showed an inconspicuous difference under 0–5% NaCl stress (*p* > 0.05), demonstrating that GXDK6 has a strong salt-tolerant ability and may need to take a while to adapt to salt stress. Moreover, the morphology of GXDK6 under NaCl stress was observed by SEM. The results showed that the cell morphology of GXDK6 was round, smooth, and oval under non-salt stress (Figure 1B). However, it showed a contracted, elongated, or rod-shaped form under 10% NaCl stress (Figure 1C), suggesting that the morphological changes in GXDK6 may respond to NaCl stress due to its regulation of lipid metabolism and membrane permeability (Wang D. et al., 2021). For testing of this hypothesis, the transcriptome and proteome of GXDK6 were further investigated under NaCl stress.

### Genome-Wide Analysis Revealed That Lipid Metabolism-Related Genes Were Significantly Enriched in Pathways Contributing to Salt-Tolerant Survival

117 genes relevant to the lipid metabolism of GXDK6 were annotated, which accounted for 8.77% of the total metabolic regulation genes (1,334 genes, provided in Supplementary Table S1; Supplementary Figure S1), indicating that GXDK6 had the ability to produce rich lipids. Particularly, mangrove-associated microorganisms resulted in characterization of almost 1,000 new metabolites, among them, ∼850 derived from fungi, and ∼120 from bacteria (Ancheeva et al., 2018; Blunt et al., 2018). Interestingly, whole-genome analysis found *M. guilliermondii* strain had abundant genes [e.g., *ERG1* encoding squalene monooxygenase, *ERG5* encoding cytochrome P450 61, *ERG6* encoding sterol 24-C-methyltransferase, *ERG9* encoding squalene synthase, *ERG11* encoding lanosterol 14-alpha demethylase, and *ERG24* encoding delta (14)-sterol reductase] regulating steroid biosynthesis pathway, which regulate the biosynthesis of sterols and terpenoids. Therefore, GXDK6 could have a specific regulatory network to biosynthesize important terpenoids. Previous studies have shown that GXDK6 had the ability to biosynthesize nerol (an acyclic monoterpene), which was widely used in food, cosmetics and pharmaceuticals as the valuable fragrance (Mo et al., 2021), aptly validating the above speculation.

In addition, these genes (e.g., *GPD* encoding glycerol-3-phosphate dehydrogenase*, ERG24*, and *GPP1*) were mainly involved in regulating glycerophospholipid metabolism, lipid biosynthesis proteins, glycerolipid metabolism, fatty acid degradation, steroid biosynthesis, sphingolipid metabolism, the biosynthesis of unsaturated fatty acids, fatty acid biosynthesis, and fatty acid elongation (Figure 1D; Table 1). Among them, glycerophospholipid metabolism, glycerolipid metabolism, fatty acid degradation, the biosynthesis of unsaturated fatty acids, fatty acid biosynthesis, and fatty acid elongation played an important role in the defense and survival of microorganisms (Chen et al., 2015). Vigorous lipid metabolism was speculated to have contributed to salt-tolerant survival. Therefore, the transcriptome and proteome of GXDK6 were further studied.

**TABLE 1:** KEGG pathway enrichment analysis of lipid metabolism in GXDK6.

Pathway	Number of genes
Fatty acid biosynthesis	11
Fatty acid elongation	9
Fatty acid degradation	19
Synthesis and degradation of ketone bodies	2
Cutin, suberine and wax biosynthesis	1
Steroid biosynthesis	15
Primary bile acid biosynthesis	1
Secondary bile acid biosynthesis	1
Steroid hormone biosynthesis	4
Glycerolipid metabolism	24
Glycerophospholipid metabolism	30
Ether lipid metabolism	5
Arachidonic acid metabolism	4
Linoleic acid metabolism	1
alpha-Linolenic acid metabolism	5
Sphingolipid metabolism	14
Lipid biosynthesis proteins	26
Biosynthesis of unsaturated fatty acids	13

### Transcriptome Analysis Revealed That ERG24 Was Significantly Down-Regulated in *M. guilliermondii* Strain After Perceived Salt Stress

**FIGURE 2:**
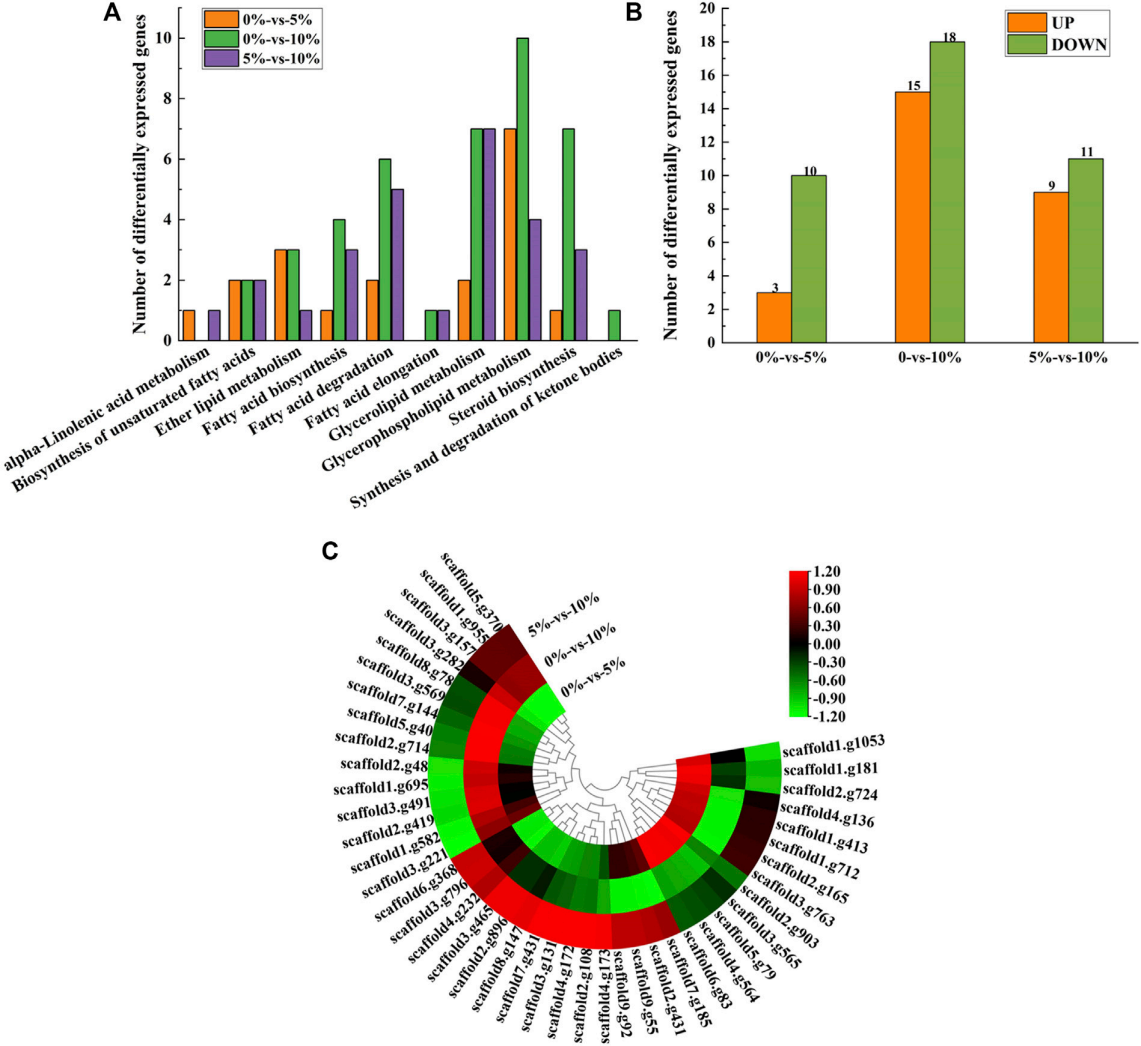
Transcriptome analysis of lipid metabolism. **(A)** Expression of differentially expressed genes at 0, 5, and 10% NaCl; **(B)** Enrichment of pathways under different NaCl stress; **(C)** Heatmap of differentially expressed genes.

As shown in Figure 2B, three genes were upregulated and 10 genes were downregulated under 5% NaCl stress compared with those under salt-free stress, while 15 genes were upregulated and 18 genes were downregulated under 10% NaCl stress. These differentially expressed genes (e.g., *GPD*, *GUT*, and *GPP*) under 10% NaCl stress were significantly enriched in glycerophospholipid metabolism (accounting for 30.30%), glycerolipid metabolism (accounting for 21.21%), steroid biosynthesis (accounting for 21.21%), fatty acid degradation (accounting for 18.18%), fatty acid biosynthesis (accounting for 12.12%), ether lipid metabolism (accounting for 9.09%), the biosynthesis of unsaturated fatty acids (accounting for 6.06%), fatty acid elongation (accounting for 3.03%), and synthesis and degradation of ketone bodies (accounting for 3.03%, Figure 2A). Numerous differentially expressed genes (e.g., *GPD*, *GUT*, and *GPP*) that regulate lipid metabolism were significantly enriched in pathways that contributed to salt-tolerant survival (e.g., glycerophospholipid metabolism, glycerolipid metabolism, fatty acid degradation) (Xia et al., 2019; Yang et al., 2019) under 10%NaCl stress, which meant *M. guilliermondii* strain had good salt resistance, providing a reference for the application in high salt environments (e.g., soy sauce brewing).

With the condition of 10% NaCl stress as an example, the most significantly upregulated gene was *GPD* (upregulated by 4.00 folds) (Figure 2C, provided in Supplementary Table S4). *GPD* belonged to the HOG-MAPK pathway, which contributed to the timely reception of signals generated by salt stimulation in *M. guilliermondii* strain and induced the expression of targeted regulatory genes in cells, thereby contributing to salt-tolerant survival (Akhtar et al., 2000). According to the analysis of glycerol metabolism, genes (e.g., *GUT*, *ALD5*, and *GPP*) were also significantly up-regulated under 10% NaCl stress (Supplementary Figure S2). It would be reasonable to speculate that *M. guilliermondii* strain regulate glycerol metabolism and accumulate to maintain intra- and extracellular osmotic pressure, thereby promoting the survival of GXDK6 under salt stress. Similar results had also been demonstrated by Duskova et al. (2015), Capusoni et al. (2019), and Lee and Levin (2015). We have also confirmed through experiments that exogenous addition of glycerol enhanced the salt tolerance of GXDK6 under stress (Supplementary Figure S3).

However, the most significantly downregulated gene was *ERG24* (downregulated by 3.15 folds, provided in Supplementary Table S4). Delta (14)-sterol reductase (regulated by *ERG24*) is a drug target of fenpropimorph, which is involved in step 2 of the subpathway that synthesizes zymosterol from lanosterol. Due to the significant down-regulation of this gene under salt stress, it was speculated that NaCl stress may change the sensitivity of *M. guilliermondii* strain to drugs. This speculation was indirectly verified by Bhuiyan et al. (2007).

In order to verify the reliability of the transcriptome data, *GUT1*, *GPP1* and *ADH7* related to glycerol metabolism were selected for RT-qPCR verification. It can be seen from Supplementary Figure S4 that the transcription levels of the three genes verified by RT-qPCR are up-regulated, which is consistent with the results of the transcriptome data, indicating the reliability of the transcriptome data. Furthermore, we analyzed the proteomic data of lipid metabolism in GXDK6.

### Proteomic Analysis Revealed That NaCl Stress Inhibited Spore Germination of *M. guilliermondii* Strain

**FIGURE 3:**
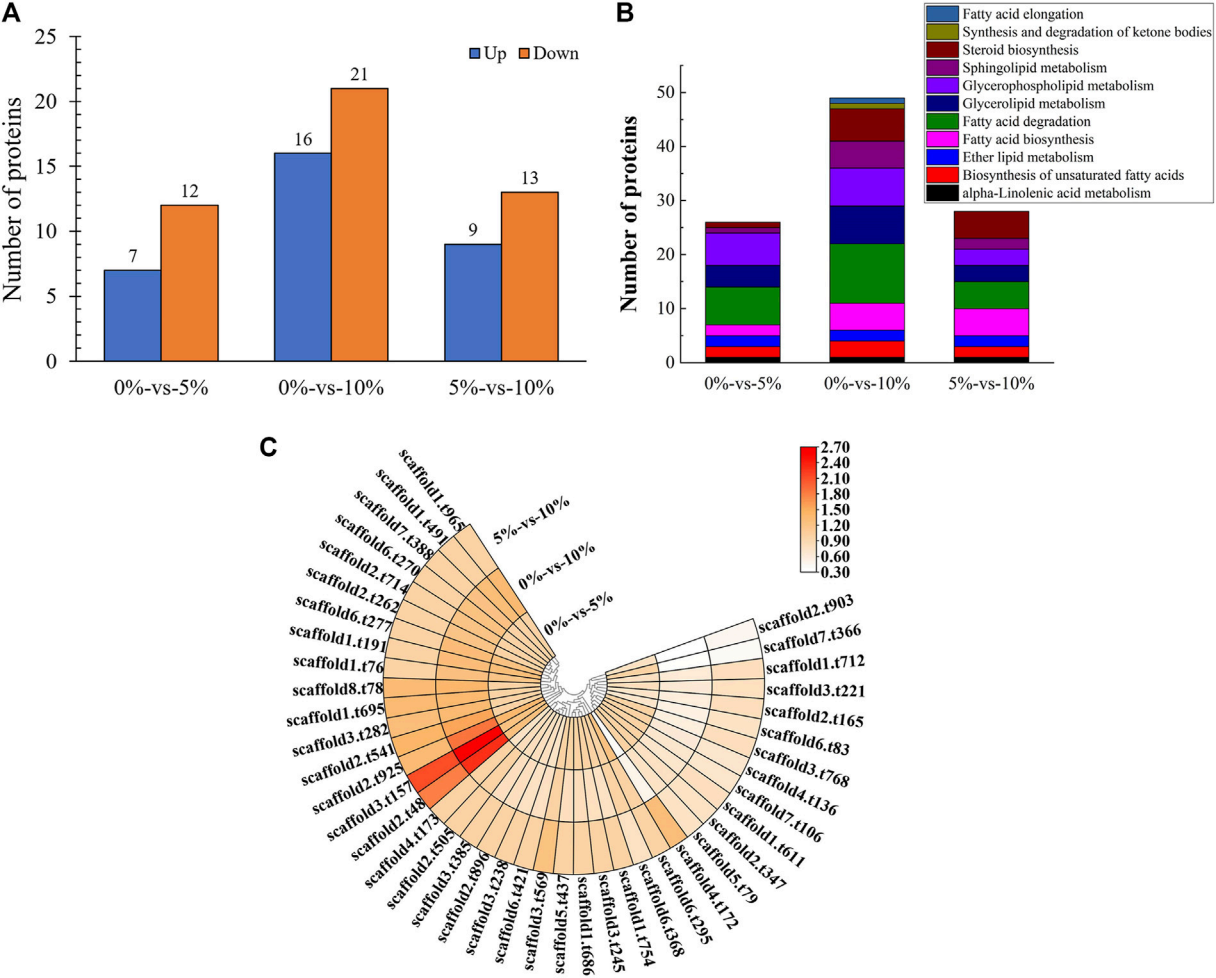
Proteomic analysis of lipid metabolism. **(A)** Number of differentially expressed proteins under different salt stress; **(B)** Pathway enrichment of differentially expressed proteins; **(C)** Differentially expressed proteins under different salt stress.

16 proteins were significantly upregulated and 21 proteins were significantly downregulated, which were all linked to lipid metabolism in GXDK6 under 10% NaCl stress (Figure 3A). These differentially expressed proteins were enriched in fatty acid degradation (accounting for 29.73%), glycerophospholipid metabolism (accounting for 18.92%), glycerolipid metabolism (accounting for 18.92%), steroid biosynthesis (accounting for 16.22%), sphingolipid metabolism (accounting for 13.51%), fatty acid biosynthesis (accounting for 13.51%), the biosynthesis of unsaturated fatty acids (accounting for 8.11%), ether lipid metabolism (accounting for 5.41%), alpha-linolenic acid metabolism (accounting for 2.70%), synthesis and degradation of ketone bodies (accounting for 2.70%), and fatty acid elongation (accounting for 2.70%, Figure 3B). Among them, the top three significantly upregulated proteins were alcohol dehydrogenase 3 (upregulated by 1.20 folds, promoting the dehydrogenation of aldehydes to produce alcohols, provided in Supplementary Table S11), aldehyde dehydrogenase 5 (upregulated by 1.39 folds, reducing the accumulation of aldehydes, provided in Supplementary Table S11), and 3-ketoacyl-thiolase peroxisomal (upregulated by 0.89 folds, a kind of peroxisomal involved in the pathway of fatty acid metabolism). The top three significantly downregulated proteins were NADPH-dependent 1-acyldihydroxyacetone phosphate reductase (downregulated by 0.98 folds; plays a role in cell wall biogenesis, but this effect may be indirect by affecting the activities of cell wall synthesis enzymes), methylsterol monooxygenase (downregulated by 1.43 folds; involved in the pathway of ergosterol biosynthesis, which is a part of steroid metabolism), and alcohol dehydrogenase 2 (downregulated by 1.47 folds; this isozyme preferentially catalyzes the conversion of ethanol to acetaldehyde and acts on various primary unbranched aliphatic alcohols) under 10% NaCl (Figure 3C, provided in Supplementary Table S11). Aldehyde dehydrogenase 5 (regulated by *ALDH5*) and alcohol dehydrogenase (regulated by *ADH2*, and *ADH3*) had functions of protecting cells and maintaining cellular osmotic pressure homeostasis.

Among them, NADPH-dependent 1-acyldihydroxyacetone phosphate reductase (regulatory gene *AYR1*, which regulated the budding and division of cell membranes) (Athenstaedt and Daum, 2000) regulated phosphatidic acid biosynthesis. According to significant down-regulated expression of this protein under high salt stress, it was reasonable to speculate that the budding division of the cell membrane was inhibited, thus inhibiting cell proliferation. This finding further revealed the reason for the decrease in the number of cells under 10% NaCl. The test showed that with the incubation time at 16 h as an example, the OD_600_ of GXDK6 decreased by 64.39% under 10% NaCl stress when compared with salt-free stress. The presence of NaCl during the cultivation process caused a slight decrease in the growth rate. This result was also confirmed by Capusoni et al. (2019) and Petrovic et al. (2002). The analysis of the growth state of *M. guilliermondii* strain under salt stress could provide a reference for its application in high-salt environments.

In addition, the methylsterol monooxygenase regulates the biosynthesis of ergosterol (Ward et al., 2018), which contributes to maintaining the rigidity of the plasma membrane (Schützhold et al., 2016), and serves as the precursor for manufacturing steroid drugs (Liu et al., 2019). However, the methylsterol monooxygenase (subcellularly located in the endoplasmic reticulum membrane) was significantly down-regulated under 10% NaCl stress. It was reasonable to speculate that *M. guilliermondii* strain can regulate the expression of membrane proteins to enhance resistance to stress environments.

### Transcriptomic and Proteomic Analysis Revealed That Lanosterol 14-Alpha Demethylase was Down-Regulated in *M. guilliermondii* Strain After Perceived Salt Stress

**FIGURE 4:**
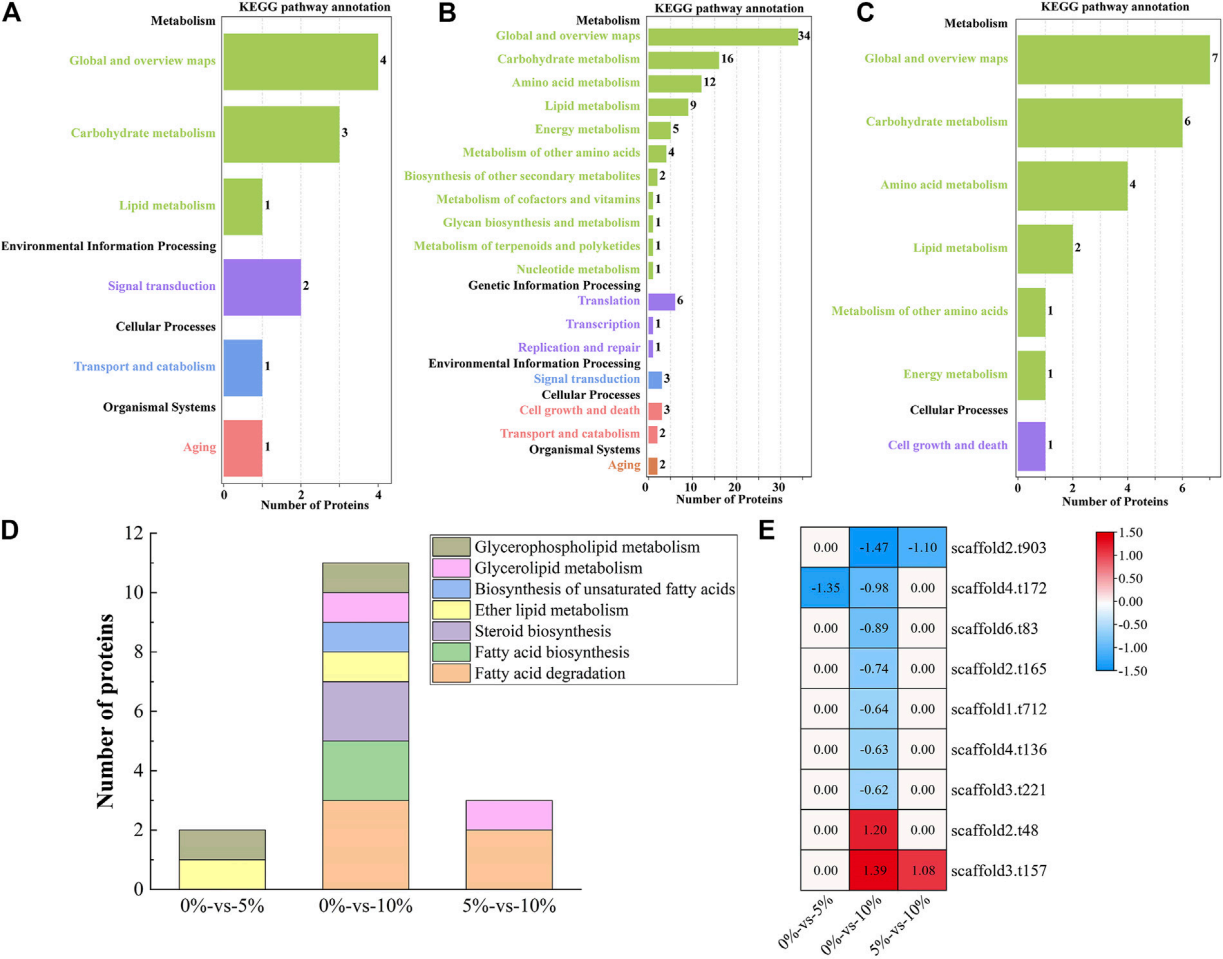
Association analysis of multi-omics data relevant to lipid metabolism. **(A)** Multi-omics association analysis under the condition of 0%-vs-5%; **(B)** Multi-omics association analysis under the condition of 0%-vs-10%; **(C)** Multi-omics association analysis under the condition of 5%-vs-10%; **(D)** KO enrichment of related differentially expressed genes and differentially expressed proteins; **(E)** Related differentially expressed genes and differentially expressed proteins.

One differentially expressed gene (*AYR1*) and one differentially expressed protein (NADPH-dependent 1-acyldihydroxyacetone phosphate reductase, which could convert acyl and alkyl dihydroxyacetone-phosphate into glycerolipids and ether lipids) were associated in the pathway of lipid metabolism under 5% NaCl compared with those under salt-free stress (Figure 4A, provided in Supplementary Table S14), while nine differentially expressed genes and nine differentially expressed proteins were associated under 10% NaCl (Figures 4B,E, provided in Supplementary Table S15). When GXDK6 was under 10% NaCl, two differentially expressed genes and two differentially expressed protein were associated in lipid metabolism compared with those under 5% NaCl stress (Figure 4C, provided in Supplementary Table S16). With the condition of 10% NaCl stress as an example, the related genes and proteins were mainly enriched in fatty acid degradation (accounting for 33.33%), fatty acid biosynthesis (accounting for 22.22%), steroid biosynthesis (accounting for 22.22%), ether lipid metabolism (accounting for 11.11%), the biosynthesis of unsaturated fatty acids (accounting for 11.11%), glycerolipid metabolism (accounting for 11.11%), and glycerophospholipid metabolism (accounting for 11.11%, Figure 4D). These differentially expressed proteins were alcohol dehydrogenase 2 (downregulated by 1.47 folds), NADPH-dependent 1-acyl dihydroxyacetone phosphate reductase (downregulated by 0.98 folds), squalene monooxygenase (downregulated by 0.89 folds), fatty acid synthase alpha subunit (downregulated by 0.74 folds), acetyl-CoA carboxylase (downregulated by 0.64 folds), lanosterol 14-alpha demethylase (downregulated by 0.63 folds), delta (12)-fatty-acid desaturase (downregulated by 0.62 folds), alcohol dehydrogenase 3 (upregulated by 1.20 folds), and aldehyde dehydrogenase (upregulated by 1.39 folds).

Squalene monooxygenase was the key rate-limiting enzyme for the synthesis of sterols and triterpenoids (Han et al., 2020). The expression of this enzyme was downregulated under 10% NaCl stress, indicating that the synthesis of sterol substances was changed under salt stress. Lanosterol 14-alpha demethylase was a key enzyme in ergosterol biosynthesis. When ergosterol synthesis was blocked, the cell membranes were damaged, thus making antifungal drugs, such as azoles, work (Kauffman and Carver, 1997; Sagatova et al., 2015). Studies have found that the protein was significantly differentially expressed under salt stress. It was speculated that the killing effect of antifungal drugs would be affected by NaCl stress. Therefore, we explored the changes of resistance in GXDK6 under salt stress.

### NaCl Stress Enhanced the Killing Effect of Fluconazole on *M. guilliermondii* Strain

**FIGURE 5:**
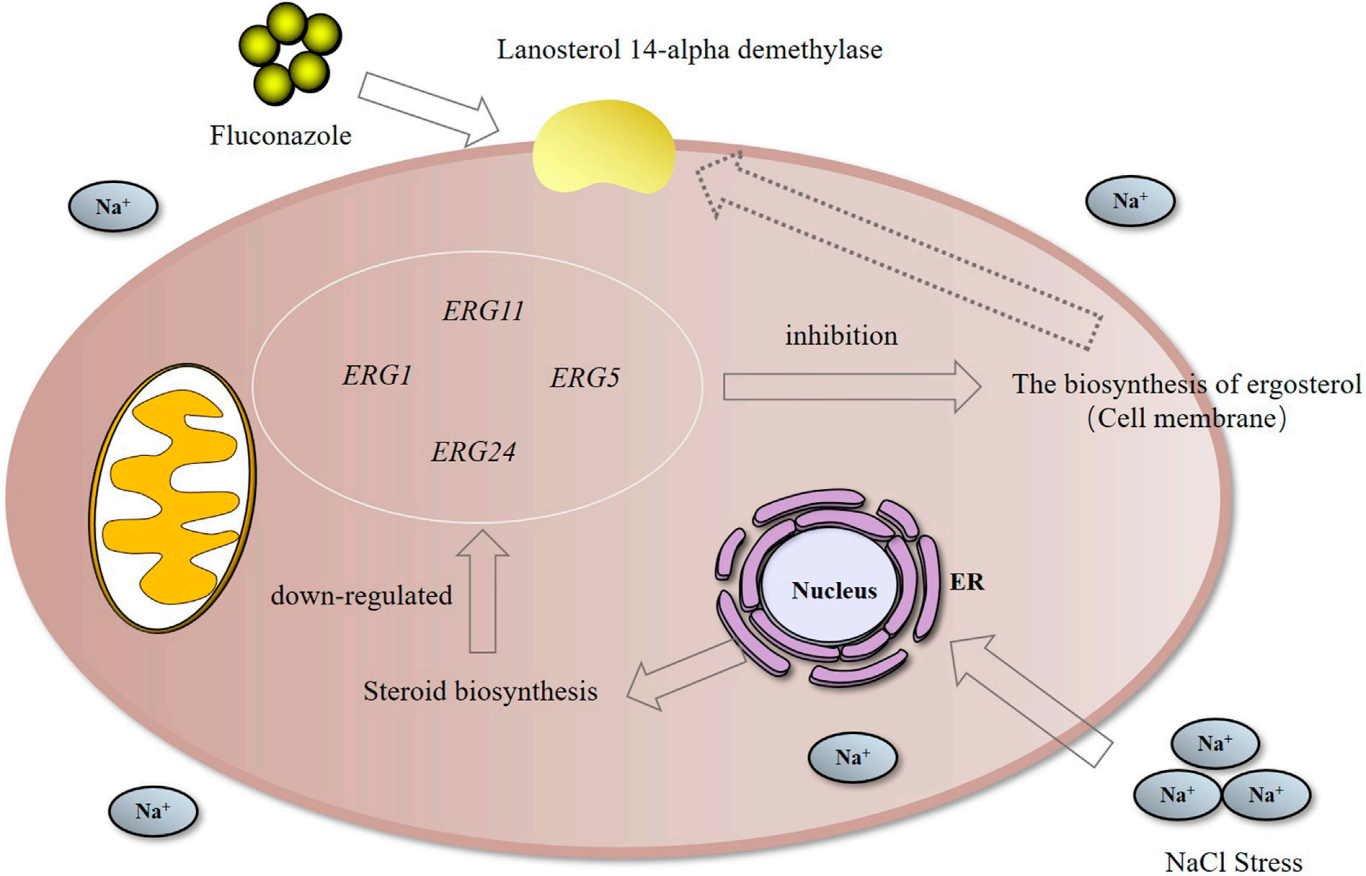
Drug resistance of GXDK6 to fluconazole under NaCl stress.

As shown in Supplementary Table S4, the differentially expressed genes were *ERG1* (downregulated by 2.34 folds, encoding squalene monooxygenase), *ERG5* (downregulated by 1.09 folds, encoding squalene monooxygenase), *ERG11* (downregulated by -1.17 folds, encoding lanosterol 14-alpha demethylase), and *ERG24* [downregulated by 3.15 folds, encoding delta (14)-sterol reductase]. The above genes were mainly involved in the biosynthesis of ergosterol (Konecna et al., 2016; Guo et al.,2018). The critical role of ergosterol in maintaining cell membrane integrity and its uniqueness in fungi make it a highly attractive antifungal drug target (Li Y. et al., 2021). Previous studies had shown that *ERG24* was an antifungal target site for azole drugs (Jia et al., 2002). Flowers et al. (2015) found that single mutations in *ERG11* (an important drug target) contributed to azole resistance in *Candida albicans*. In this study, these genes were significantly downregulated under salt stress. Therefore, we speculated that ergosterol, as a target of antifungal drugs, its synthesis pathway was inhibited under salt stress, leading to improve killing effect of fluconazole against *M. guilliermondii* strain (Long et al., 2017). This speculation was also confirmed in the present work, and the results were shown in Figure 5. The OD_600_ of GXDK6 under 10% NaCl and contained 64 μg/ml fluconazole was 0.319. However, it decreased to 0.124 under 64 μg/ml fluconazole, which was 61.13% lower than the condition of salt-free but contained 64 μg/ml fluconazole (provided in Supplementary Figure S5). Moreover, the number of colonies of GXDK6 under 10% NaCl and without fluconazole were 9.0*10^5^ cfu/ml (provided in Supplementary Figure S6). However, it decreased to 2.3*10^4^ cfu/ml under 64 μg/ml fluconazole, which was 97.70% lower than the condition of salt-free but contained 64 μg/ml fluconazole (number of colonies = 1.0*10^6^ cfu/ml, Table 2). It was considered that the genes regulating synthesis of ergosterol were down-regulated under salt stress, which lead to down-regulation of the corresponding proteins expression, thereby weakening the synthesis of ergosterol. The results demonstrated that salt stress promoted the fungicidal effect of fluconazole, which provided a new approach to enhance the killing effect of fluconazole on fungi.

**TABLE 2:** Drug resistance of GXDK6 to fluconazole under NaCl stress.

Conditions	Dilution times	Cfu/ml
0%NaCl+0 μg/ml fluconazole	10^–6^	2.1*10^8^
5%NaCl+0 μg/ml fluconazole	10^–6^	1.0*10^7^
10%NaCl+0 μg/ml fluconazole	10^–5^	9.0*10^5^
0%NaCl+64 μg/ml fluconazole	10^–5^	1.0*10^6^
5%NaCl+64 μg/ml fluconazole	10^–3^	1.1*10^5^
10%NaCl+64 μg/ml fluconazole	10^–2^	2.3*10^4^

## Conclusion

The regulation mechanism of lipid metabolism in *M. guilliermondii GXDK6* was revealed by integrative omics technology. The key genes and proteins that regulated steroid biosynthesis and glycerolipid metabolism under salt stress were identified. High-concentration salt stress could change the biosynthesis of ergosterol and inhibited cell proliferation. Further results demonstrated that the killing effect of fluconazole on *M. guilliermondii* GXDK6 was significantly enhanced under salt stimulation. Final results showed that salt stress disturbed lipid metabolism, which reduced the expression of antifungal drugs targets, thus making *M. guilliermondii* strain more sensitive to fluconazole. This work revealed the regulatory changes of lipid metabolism in *M. guilliermondii* strain under salt stress, which also provided a new strategy to enhance the fungicidal effect of antifungal drugs.

## Data Availability

The datasets presented in this study can be found in online repositories. The names of the repository/repositories and accession number(s) can be found in the article/Supplementary Material.
