# Supplementary material for: Multi-Omics Analysis of Lipid Metabolism for a Marine Probiotic Meyerozyma guilliermondii GXDK6 Under High NaCl Stress
Source: Front Genet. 2022 Jan 13;12:798535. doi: 10.3389/fgene.2021.798535 (PMC8792971; doi:10.3389/fgene.2021.798535)

**Supplementary materials**

**Supplementary Table S1**: The genes of lipid metabolism in GXDK6.

| Number | orf_name | gene name | Class B |
| --- | --- | --- | --- |
| 1 | scaffold1.g43 | *CER10* | Lipid metabolism |
| 2 | scaffold1.g76 | *PECI* | Lipid metabolism |
| 3 | scaffold1.g191 | *DAK* | Lipid metabolism |
| 4 | scaffold1.g349 | *CLD1* | Lipid metabolism |
| 5 | scaffold1.g371 | *LPT1* | Lipid metabolism |
| 6 | scaffold1.g405 | *SOAT* | Lipid metabolism |
| 7 | scaffold1.g430 | *SPT* | Lipid metabolism |
| 8 | scaffold1.g528 | *SGPL1* | Lipid metabolism |
| 9 | scaffold1.g567 | *ACOX1* | Lipid metabolism |
| 10 | scaffold1.g686 | *ACADM* | Lipid metabolism |
| 11 | scaffold1.g695 | *EPT1* | Lipid metabolism |
| 12 | scaffold1.g712 | *ACACA* | Lipid metabolism |
| 13 | scaffold1.g754 | *LIPA* | Lipid metabolism |
| 14 | scaffold1.g761 | *AYR1* | Lipid metabolism |
| 15 | scaffold1.g792 | *ATG15* | Lipid metabolism |
| 16 | scaffold1.g955 | *glpK* | Lipid metabolism |
| 17 | scaffold1.g968 | *CRLS* | Lipid metabolism |
| 18 | scaffold1.g1052 | *aslA* | Lipid metabolism |
| 19 | scaffold1.g1053 | *ACOX1* | Lipid metabolism |
| 20 | scaffold2.g48 | *adhP* | Lipid metabolism |
| 21 | scaffold2.g51 | *GCY1* | Lipid metabolism |
| 22 | scaffold2.g77 | *CKI1* | Lipid metabolism |
| 23 | scaffold2.g165 | *FAS2* | Lipid metabolism |
| 24 | scaffold2.g184 | *PCYT2* | Lipid metabolism |
| 25 | scaffold2.g262 | *LAG1* | Lipid metabolism |
| 26 | scaffold2.g347 | *MESO1* | Lipid metabolism |
| 27 | scaffold2.g387 | *MECR* | Lipid metabolism |
| 28 | scaffold2.g419 | *fabF* | Lipid metabolism |
| 29 | scaffold2.g483 | *ACSL* | Lipid metabolism |
| 30 | scaffold2.g505 | *SPHK* | Lipid metabolism |
| 31 | scaffold2.g510 | *hdhA* | Lipid metabolism |
| 32 | scaffold2.g541 | *ACSL* | Lipid metabolism |
| 33 | scaffold2.g678 | *gpx* | Lipid metabolism |
| 34 | scaffold2.g685 | *GPD1* | Lipid metabolism |
| 35 | scaffold2.g714 | *atoB* | Lipid metabolism |
| 36 | scaffold2.g724 | *adhP* | Lipid metabolism |
| 37 | scaffold2.g744 | *PPT* | Lipid metabolism |
| 38 | scaffold2.g886 | *ACAA1* | Lipid metabolism |
| 39 | scaffold2.g896 | *PLB* | Lipid metabolism |
| 40 | scaffold2.g903 | *adhP* | Lipid metabolism |
| 41 | scaffold2.g925 | *ACAA1* | Lipid metabolism |
| 42 | scaffold3.g108 | *GDE1* | Lipid metabolism |
| 43 | scaffold3.g131 | *psd* | Lipid metabolism |
| 44 | scaffold3.g157 | *ALDH* | Lipid metabolism |
| 45 | scaffold3.g212 | *CLD1* | Lipid metabolism |
| 46 | scaffold3.g225 | *ELO3* | Lipid metabolism |
| 47 | scaffold3.g238 | *ADH5* | Lipid metabolism |
| 48 | scaffold3.g239 | *ADH5* | Lipid metabolism |
| 49 | scaffold3.g245 | *ACSL* | Lipid metabolism |
| 50 | scaffold3.g282 | *GLYK* | Lipid metabolism |
| 51 | scaffold3.g320 | *CDS1* | Lipid metabolism |
| 52 | scaffold3.g358 | *ELO2* | Lipid metabolism |
| 53 | scaffold3.g385 | *ALDH* | Lipid metabolism |
| 54 | scaffold3.g471 | *TSC10* | Lipid metabolism |
| 55 | scaffold3.g497 | *TGL4* | Lipid metabolism |
| 56 | scaffold3.g565 | *ERG24* | Lipid metabolism |
| 57 | scaffold3.g569 | *TAZ* | Lipid metabolism |
| 58 | scaffold3.g581 | *PCYT1* | Lipid metabolism |
| 59 | scaffold3.g600 | *TGL3* | Lipid metabolism |
| 60 | scaffold3.g732 | *LTA4H* | Lipid metabolism |
| 61 | scaffold3.g768 | *ACER3* | Lipid metabolism |
| 62 | scaffold3.g796 | *GPP1* | Lipid metabolism |
| 63 | scaffold3.g830 | *fabG* | Lipid metabolism |
| 64 | scaffold4.g45 | *DPP1* | Lipid metabolism |
| 65 | scaffold4.g136 | *CYP51* | Lipid metabolism |
| 66 | scaffold4.g172 | *HSD17B2* | Lipid metabolism |
| 67 | scaffold4.g173 | *AYR1* | Lipid metabolism |
| 68 | scaffold4.g226 | *ERG4* | Lipid metabolism |
| 69 | scaffold4.g232 | *GAT* | Lipid metabolism |
| 70 | scaffold4.g291 | *fabG* | Lipid metabolism |
| 71 | scaffold4.g357 | *ACOT8* | Lipid metabolism |
| 72 | scaffold4.g445 | *ACOT9* | Lipid metabolism |
| 73 | scaffold4.g564 | *PHS1* | Lipid metabolism |
| 74 | scaffold4.g565 | *PHS1* | Lipid metabolism |
| 75 | scaffold5.g40 | *FDFT1* | Lipid metabolism |
| 76 | scaffold5.g79 | *ERG5* | Lipid metabolism |
| 77 | scaffold5.g84 | *LAG1* | Lipid metabolism |
| 78 | scaffold5.g90 | *ERG27* | Lipid metabolism |
| 79 | scaffold5.g93 | *SMPD2* | Lipid metabolism |
| 80 | scaffold5.g236 | *galA* | Lipid metabolism |
| 81 | scaffold5.g299 | *ACSS* | Lipid metabolism |
| 82 | scaffold5.g321 | *KAR* | Lipid metabolism |
| 83 | scaffold5.g403 | *ERG2* | Lipid metabolism |
| 84 | scaffold5.g407 | *LRO1* | Lipid metabolism |
| 85 | scaffold5.g412 | *ACSL* | Lipid metabolism |
| 86 | scaffold5.g437 | *AGPAT1_2* | Lipid metabolism |
| 87 | scaffold6.g45 | *LTA4H* | Lipid metabolism |
| 88 | scaffold6.g83 | *ERG1;* | Lipid metabolism |
| 89 | scaffold6.g169 | *DEGS* | Lipid metabolism |
| 90 | scaffold6.g193 | *pgsA* | Lipid metabolism |
| 91 | scaffold6.g228 | *hcs1* | Lipid metabolism |
| 92 | scaffold6.g233 | *PEMT* | Lipid metabolism |
| 93 | scaffold6.g270 | *GEP4* | Lipid metabolism |
| 94 | scaffold6.g276 | *ETNK* | Lipid metabolism |
| 95 | scaffold6.g277 | *DPP1* | Lipid metabolism |
| 96 | scaffold6.g295 | *CDIPT* | Lipid metabolism |
| 97 | scaffold6.g368 | *fabD* | Lipid metabolism |
| 98 | scaffold6.g421 | *CHO2* | Lipid metabolism |
| 99 | scaffold6.g444 | *CYP86A1* | Lipid metabolism |
| 100 | scaffold7.g17 | *TGL2* | Lipid metabolism |
| 101 | scaffold7.g89 | *ACSS* | Lipid metabolism |
| 102 | scaffold7.g106 | *SUR2* | Lipid metabolism |
| 103 | scaffold7.g108 | *GAT* | Lipid metabolism |
| 104 | scaffold7.g161 | *glpA* | Lipid metabolism |
| 105 | scaffold7.g185 | *ALDH* | Lipid metabolism |
| 106 | scaffold7.g244 | *fabG* | Lipid metabolism |
| 107 | scaffold7.g301 | *MECR* | Lipid metabolism |
| 108 | scaffold7.g303 | *ERG7* | Lipid metabolism |
| 109 | scaffold7.g388 | *UGCG* | Lipid metabolism |
| 110 | scaffold7.g431 | *adh* | Lipid metabolism |
| 111 | scaffold8.g78 | *adh* | Lipid metabolism |
| 112 | scaffold8.g81 | *PGC1* | Lipid metabolism |
| 113 | scaffold8.g127 | *COMT* | Lipid metabolism |
| 114 | scaffold8.g153 | *MGLL* | Lipid metabolism |
| 115 | scaffold8.g177 | *ERG26* | Lipid metabolism |
| 116 | scaffold9.g12 | *galA* | Lipid metabolism |
| 117 | scaffold9.g55 | *ERG24* | Lipid metabolism |

**Supplementary Table S2**: The expression of differential genes in lipid metabolism at 0%, 5%, and 10% NaCl.

| Conditions | UP | DOWN |
| --- | --- | --- |
| 0%-vs-5% | 3 | 10 |
| 0-vs-10% | 15 | 18 |
| 5%-vs-10% | 9 | 11 |

**Supplementary Table S3**: Enrichment of differential genes in lipid metabolism under salt stress.

| Pathway | 0%-vs-5% | 0-vs-10% | 5%-vs-10% |
| --- | --- | --- | --- |
| alpha-Linolenic acid metabolism | 1 | 0 | 1 |
| Biosynthesis of unsaturated fatty acids | 2 | 2 | 2 |
| Ether lipid metabolism | 3 | 3 | 1 |
| Fatty acid biosynthesis | 1 | 4 | 3 |
| Fatty acid degradation | 2 | 6 | 5 |
| Fatty acid elongation | 0 | 1 | 1 |
| Glycerolipid metabolism | 2 | 7 | 7 |
| Glycerophospholipid metabolism | 7 | 10 | 4 |
| Steroid biosynthesis | 1 | 7 | 3 |
| Synthesis and degradation of ketone bodies | 0 | 1 | 0 |

**Supplementary Table S4**: Differential genes of lipid metabolism.

| ID | Symbol | A-vs-B-log2fc | A-vs-B-pvalue | A-vs-B-fdr | A-vs-C-log2fc | A-vs-C-pvalue | A-vs-C-fdr | B-vs-C-log2fc | B-vs-C-pvalue | B-vs-C-fdr |
| --- | --- | --- | --- | --- | --- | --- | --- | --- | --- | --- |
| scaffold1.g1053 | *POX1* | 1.1889 | 0.0000 | 0.0000 | 0.0327 | 0.4802 | 0.5788 | -1.1562 | 0.0000 | 0.0000 |
| scaffold2.g108 | *FAS1* | -2.4111 | 0.0000 | 0.0000 | -2.3000 | 0.0000 | 0.0000 | 0.1111 | 0.2307 | 0.3377 |
| scaffold2.g896 | *PLB3* | -1.2443 | 0.0000 | 0.0000 | -0.6070 | 0.0000 | 0.0000 | 0.6372 | 0.0006 | 0.0020 |
| scaffold3.g131 | *PSD2* | -1.6139 | 0.0001 | 0.0008 | -1.3522 | 0.0001 | 0.0002 | 0.2617 | 0.9337 | 0.9521 |
| scaffold3.g221 | *FAH12* | 1.1097 | 0.0000 | 0.0000 | 1.2126 | 0.0000 | 0.0000 | 0.1030 | 0.1071 | 0.1825 |
| scaffold3.g465 | *NTE1* | -1.4806 | 0.0000 | 0.0002 | -0.7362 | 0.0029 | 0.0069 | 0.7443 | 0.0504 | 0.0974 |
| scaffold4.g172 | *AYR1* | -2.7602 | 0.0000 | 0.0000 | -2.6886 | 0.0000 | 0.0000 | 0.0716 | 0.2719 | 0.3838 |
| scaffold4.g173 | *AYR1* | -1.0535 | 0.0005 | 0.0024 | -1.2761 | 0.0000 | 0.0000 | -0.2227 | 0.0738 | 0.1339 |
| scaffold7.g144 | *GPD* | 1.8755 | 0.0000 | 0.0000 | 3.9986 | 0.0000 | 0.0000 | 2.1231 | 0.0000 | 0.0000 |
| scaffold7.g431 | *ADH6* | -1.2829 | 0.0162 | 0.0446 | -0.9894 | 0.0147 | 0.0297 | 0.2935 | 0.9255 | 0.9464 |
| scaffold8.g147 | *SPO14* | -1.6684 | 0.0000 | 0.0000 | -0.5335 | 0.0068 | 0.0150 | 1.1349 | 0.0000 | 0.0000 |
| scaffold9.g55 | *ERG24* | -1.8757 | 0.0000 | 0.0000 | -3.1504 | 0.0000 | 0.0000 | -1.2747 | 0.0000 | 0.0000 |
| scaffold9.g92 | *aldA* | -1.5290 | 0.0000 | 0.0000 | -2.5552 | 0.0000 | 0.0000 | -1.0262 | 0.0000 | 0.0002 |
| scaffold1.g413 | *--* | -0.4440 | 0.3014 | 0.4343 | -1.1275 | 0.0004 | 0.0013 | -0.6835 | 0.0021 | 0.0059 |
| scaffold1.g582 | *ERG6* | 0.8381 | 0.0000 | 0.0000 | 1.0513 | 0.0000 | 0.0000 | 0.2131 | 0.6678 | 0.7563 |
| scaffold1.g695 | *EPT1* | 0.7697 | 0.0000 | 0.0000 | 1.0478 | 0.0000 | 0.0000 | 0.2781 | 0.8813 | 0.9148 |
| scaffold1.g712 | *ACC1* | -0.8917 | 0.0000 | 0.0003 | -2.2402 | 0.0000 | 0.0000 | -1.3485 | 0.0000 | 0.0000 |
| scaffold1.g955 | *GUT1* | 0.1413 | 0.0659 | 0.1349 | 1.2397 | 0.0000 | 0.0000 | 1.0984 | 0.0000 | 0.0000 |
| scaffold2.g165 | *FAS2* | -0.8798 | 0.0000 | 0.0000 | -2.1329 | 0.0000 | 0.0000 | -1.2531 | 0.0000 | 0.0000 |
| scaffold2.g419 | *SPBC887.13c* | 0.6922 | 0.0000 | 0.0000 | 1.0479 | 0.0000 | 0.0000 | 0.3557 | 0.4466 | 0.5591 |
| scaffold2.g431 | *SPAC6G10.03c* | -0.8503 | 0.0169 | 0.0462 | -1.4811 | 0.0000 | 0.0000 | -0.6308 | 0.0018 | 0.0051 |
| scaffold2.g48 | *ADH2* | 0.8395 | 0.0000 | 0.0000 | 1.2041 | 0.0000 | 0.0000 | 0.3646 | 0.5615 | 0.6645 |
| scaffold2.g714 | *PACTA* | 0.5693 | 0.0000 | 0.0000 | 1.1210 | 0.0000 | 0.0000 | 0.5517 | 0.0380 | 0.0765 |
| scaffold2.g903 | *ADH2* | -0.0534 | 0.8025 | 0.8697 | -1.4765 | 0.0000 | 0.0000 | -1.4230 | 0.0000 | 0.0000 |
| scaffold3.g157 | *ALD5* | 0.1607 | 0.0969 | 0.1821 | 1.6809 | 0.0000 | 0.0000 | 1.5202 | 0.0000 | 0.0000 |
| scaffold3.g282 | *TDA10* | 0.6002 | 0.2881 | 0.4190 | 2.0815 | 0.0002 | 0.0006 | 1.4813 | 0.0011 | 0.0033 |
| scaffold3.g491 | *GPP1* | 0.8764 | 0.0000 | 0.0000 | 1.2927 | 0.0000 | 0.0000 | 0.4163 | 0.1829 | 0.2808 |
| scaffold3.g565 | *ERG24* | -0.4972 | 0.0012 | 0.0049 | -1.9847 | 0.0000 | 0.0000 | -1.4875 | 0.0000 | 0.0000 |
| scaffold3.g569 | *TAZ1* | 0.5807 | 0.0003 | 0.0015 | 1.3329 | 0.0000 | 0.0000 | 0.7521 | 0.0001 | 0.0003 |
| scaffold3.g763 | *CHO1* | -0.4250 | 0.0170 | 0.0465 | -1.0120 | 0.0000 | 0.0000 | -0.5869 | 0.0000 | 0.0000 |
| scaffold4.g136 | *ERG11* | -0.4170 | 0.0046 | 0.0159 | -1.1662 | 0.0000 | 0.0000 | -0.7493 | 0.0000 | 0.0000 |
| scaffold4.g564 | *HACD2* | -0.2286 | 0.5182 | 0.6450 | -1.3089 | 0.0000 | 0.0000 | -1.0803 | 0.0000 | 0.0000 |
| scaffold5.g370 | *PSD1* | 0.1555 | 0.1082 | 0.1985 | 1.2330 | 0.0000 | 0.0000 | 1.0775 | 0.0000 | 0.0000 |
| scaffold5.g40 | *ERG9* | 0.6514 | 0.0000 | 0.0000 | 1.2990 | 0.0000 | 0.0000 | 0.6475 | 0.0006 | 0.0019 |
| scaffold5.g79 | *ERG5* | -0.2206 | 0.5152 | 0.6427 | -1.0871 | 0.0000 | 0.0000 | -0.8665 | 0.0000 | 0.0000 |
| scaffold6.g83 | *ERG1* | -0.3432 | 0.1372 | 0.2366 | -2.3432 | 0.0000 | 0.0000 | -2.0000 | 0.0000 | 0.0000 |
| scaffold7.g185 | *HFD1* | -0.9599 | 0.0000 | 0.0000 | -1.7790 | 0.0000 | 0.0000 | -0.8191 | 0.0000 | 0.0000 |
| scaffold8.g78 | *ADH7* | 0.6722 | 0.0000 | 0.0000 | 1.5312 | 0.0000 | 0.0000 | 0.8590 | 0.0000 | 0.0000 |
| scaffold1.g181 | *YPR1* | 0.5653 | 0.0050 | 0.0170 | -0.9481 | 0.0002 | 0.0005 | -1.5134 | 0.0000 | 0.0000 |
| scaffold2.g724 | *adh* | 0.5364 | 0.0000 | 0.0002 | -0.4979 | 0.0000 | 0.0001 | -1.0342 | 0.0000 | 0.0000 |
| scaffold3.g796 | *GPP1* | -0.8383 | 0.0000 | 0.0000 | 0.3000 | 0.2055 | 0.2897 | 1.1384 | 0.0000 | 0.0000 |
| scaffold4.g232 | *SCT1* | -0.4809 | 0.1032 | 0.1911 | 0.8278 | 0.0005 | 0.0014 | 1.3087 | 0.0000 | 0.0000 |
| scaffold6.g368 | *fabD* | -1.2283 | 0.0662 | 0.1353 | 0.6759 | 0.2300 | 0.3182 | 1.9041 | 0.0001 | 0.0005 |

**Supplementary Table S5**: Regulation genes and proteins of glycerol metabolism.

| ID | gene name | Regulated proteins |
| --- | --- | --- |
| scaffold7.g431 | *ADH6* | alcohol dehydrogenase (NADP+) [EC:1.1.1.2] |
| scaffold9.g92 | *aldA* | aldehyde dehydrogenase (NAD+) [EC:1.2.1.3] |
| scaffold1.g955 | *GUT1* | glycerol kinase [EC:2.7.1.30] |
| scaffold3.g157 | *ALD5* | aldehyde dehydrogenase (NAD+) [EC:1.2.1.3] |
| scaffold3.g282 | *TDA10* | D-glycerate 3-kinase [EC:2.7.1.31] |
| scaffold3.g491 | *GPP1* | glycerol-1-phosphatase [EC:3.1.3.21] |
| scaffold7.g185 | *HFD1* | aldehyde dehydrogenase (NAD+) [EC:1.2.1.3] |
| scaffold8.g78 | *ADH7* | alcohol dehydrogenase (NADP+) [EC:1.1.1.2] |
| scaffold1.g181 | *YPR1* | glycerol 2-dehydrogenase (NADP+) [EC:1.1.1.156] |
| scaffold3.g796 | *GPP1* | glycerol-1-phosphatase [EC:3.1.3.21] |
| scaffold4.g232 | *SCT1* | glycerol-3-phosphate O-acyltransferase 1/2 [EC:2.3.1.15] |

**Supplementary Table S6**: Regulation genes of glycerol metabolism under 0%-vs- 5% NaCl stress.

| Id | Symbol | 0%-vs-5%-log2fc | 0%-vs-5%-pvalue | 0%-vs-5%-fdr |
| --- | --- | --- | --- | --- |
| scaffold7.g431 | *ADH6* | -1.2829 | 0.0162 | 0.0446 |
| scaffold9.g92 | *aldA* | -1.5290 | 0.0000 | 0.0000 |

**Supplementary Table S7**: Regulation genes of glycerol metabolism under 0%-vs- 10% NaCl stress.

| Id | Symbol | 0%-vs-10%-log2fc | 0%-vs-10%-pvalue | 0%-vs-10%-fdr |
| --- | --- | --- | --- | --- |
| scaffold1.g955 | *GUT1* | 1.2397 | 0.0000 | 0.0000 |
| scaffold3.g157 | *ALD5* | 1.6809 | 0.0000 | 0.0000 |
| scaffold3.g282 | *TDA10* | 2.0815 | 0.0002 | 0.0006 |
| scaffold3.g491 | *GPP1* | 1.2927 | 0.0000 | 0.0000 |
| scaffold7.g185 | *HFD1* | -1.7790 | 0.0000 | 0.0000 |
| scaffold8.g78 | *ADH7* | 1.5312 | 0.0000 | 0.0000 |
| scaffold9.g92 | *aldA* | -2.5552 | 0.0000 | 0.0000 |

**Supplementary Table S8**: Regulation genes of glycerol metabolism under 5%-vs- 10% NaCl stress.

| id | Symbol | 5%-vs-10%-log2fc | 5%-vs-10%-pvalue | 5%-vs-10%-fdr |
| --- | --- | --- | --- | --- |
| scaffold1.g181 | *YPR1* | -1.5134 | 0.0000 | 0.0000 |
| scaffold1.g955 | *GUT1* | 1.0984 | 0.0000 | 0.0000 |
| scaffold3.g157 | *ALD5* | 1.5202 | 0.0000 | 0.0000 |
| scaffold3.g282 | *TDA10* | 1.4813 | 0.0011 | 0.0033 |
| scaffold3.g796 | *GPP1* | 1.1384 | 0.0000 | 0.0000 |
| scaffold4.g232 | *SCT1* | 1.3087 | 0.0000 | 0.0000 |
| scaffold9.g92 | *aldA* | -1.0262 | 0.0000 | 0.0002 |

**Supplementary Table S9**: The expression of differential proteins in lipid metabolism at 0%, 5%, and 10% NaCl.

| Conditions | UP | DOWN |
| --- | --- | --- |
| 0%-vs-5% | 7 | 12 |
| 0-vs-10% | 16 | 21 |
| 5%-vs-10% | 9 | 13 |

**Supplementary Table S10**: Differential proteins under 0%-vs- 5% NaCl stress.

| Accession | Description | 0%-vs-5%- log2fc | P value | Regulation |
| --- | --- | --- | --- | --- |
| scaffold4.t172 | *AYR1*_NADPH-dependent 1-acyldihydroxyacetone phosphate reductase | -1.3505 | 0.0000 | DOWN |
| scaffold6.t83 | *ERG1*_Squalene monooxygenase | -0.4737 | 0.0001 | DOWN |
| scaffold2.t165 | *FAS2*_Fatty acid synthase subunit alpha | -0.4412 | 0.0000 | DOWN |
| scaffold6.t421 | *CHO2*_Phosphatidylethanolamine N-methyltransferase | -0.4168 | 0.0035 | DOWN |
| scaffold4.t173 | *AYR1*_NADPH-dependent 1-acyldihydroxyacetone phosphate reductase | -0.4016 | 0.0044 | DOWN |
| scaffold2.t903 | *ADH2*_Alcohol dehydrogenase 2 | -0.3706 | 0.0003 | DOWN |
| scaffold1.t712 | *ACAC*_Acetyl- carboxylase | -0.3474 | 0.0000 | DOWN |
| scaffold3.t238 | *FADH*_glutathione dehydrogenase | -0.3322 | 0.0001 | DOWN |
| scaffold2.t896 | *PLB3*_Lysophospholipase 3 | -0.3117 | 0.0004 | DOWN |
| scaffold3.t385 | *HFD1*_Fatty aldehyde dehydrogenase | -0.2957 | 0.0001 | DOWN |
| scaffold3.t221 | *FAH12*_Oleate hydroxylase | -0.2841 | 0.0014 | DOWN |
| scaffold2.t505 | *LCB4*_Sphingoid long chain base kinase 4 | -0.2690 | 0.0027 | DOWN |
| scaffold1.t76 | *ECI1*_3,2-trans-enoyl- isomerase | 0.2662 | 0.0033 | UP |
| scaffold3.t157 | *ALDH5*_Aldehyde dehydrogenase | 0.3064 | 0.0038 | UP |
| scaffold6.t295 | *PIS*_CDP-diacylglycerol--inositol 3-phosphatidyltransferase | 0.3107 | 0.0022 | UP |
| scaffold6.t277 | *DPP1*_Diacylglycerol pyrophosphate phosphatase 1 | 0.3176 | 0.0387 | UP |
| scaffold2.t48 | *ADH3*_Alcohol dehydrogenase | 0.3505 | 0.0166 | UP |
| scaffold1.t191 | *DAK*_Dihydroxyacetone kinase | 0.3804 | 0.0000 | UP |
| scaffold2.t925 | *THIKB*_3-ketoacyl- thiolase peroxisomal | 0.4701 | 0.0006 | UP |

**Supplementary Table S11**: Differential proteins under 0%-vs- 10% NaCl stress.

| Accession | Description | 0%-vs-10%-log2(fc) | P value | Regulation |
| --- | --- | --- | --- | --- |
| scaffold1.t191 | *DAK*_Dihydroxyacetone kinase | 0.3942 | 0.0014 | Up |
| scaffold1.t491 | *DS1P2*_Dihydrosphingosine 1-phosphate phosphatase | 0.3845 | 0.0186 | Up |
| scaffold1.t611 | *SDR1*_Short-chain type dehydrogenase reductase | -0.4783 | 0.0007 | Down |
| scaffold1.t686 | *ACD11*_Acyl- dehydrogenase family member 11 | -0.2693 | 0.0011 | Down |
| scaffold1.t695 | *EPT1*_Choline ethanolaminephosphotransferase 1 | 0.4593 | 0.0016 | Up |
| scaffold1.t712 | *ACAC*_Acetyl- carboxylase | -0.6435 | 0.0000 | Down |
| scaffold1.t754 | *TGL1*_Sterol esterase TGL1 | -0.3586 | 0.0133 | Down |
| scaffold1.t76 | *ECI1*_3,2-trans-enoyl- isomerase | 0.3387 | 0.0011 | Up |
| scaffold1.t965 | *HACD*_Very-long-chain (3R)-3-hydroxyacyl- dehydratase PASTICCINO 2 | 0.4489 | 0.0045 | Up |
| scaffold2.t165 | *FAS2*_Fatty acid synthase subunit alpha | -0.7365 | 0.0000 | Down |
| scaffold2.t262 | *LAG1*_Sphingosine N-acyltransferase lag1 | 0.2808 | 0.0017 | Up |
| scaffold2.t347 | *MSMO*_Methylsterol monooxygenase | -0.3549 | 0.0069 | Down |
| scaffold2.t48 | *ADH3*_Alcohol dehydrogenase 3 | 1.1964 | 0.0082 | Up |
| scaffold2.t541 | *LCF2*_Long-chain-fatty-acid-- ligase 2 | 0.6420 | 0.0000 | Up |
| scaffold2.t714 | *THIA*_Acetyl- acetyltransferase IA | 0.2972 | 0.0005 | Up |
| scaffold2.t896 | *PLB3*_Lysophospholipase 3 | -0.3206 | 0.0013 | Down |
| scaffold2.t903 | *ADH2*_Alcohol dehydrogenase 2 | -1.4699 | 0.0000 | Down |
| scaffold2.t925 | *THIKB*_3-ketoacyl- thiolase peroxisomal | 0.8942 | 0.0000 | Up |
| scaffold3.t157 | *ALDH5*_Aldehyde dehydrogenase 5 | 1.3866 | 0.0000 | Up |
| scaffold3.t221 | *FAH12*_Oleate hydroxylase | -0.6211 | 0.0001 | Down |
| scaffold3.t238 | *FADH*_S-(hydroxymethyl)glutathione dehydrogenase | -0.3658 | 0.0002 | Down |
| scaffold3.t245 | *LCF1*_Long-chain-fatty-acid-- ligase 1 | -0.3346 | 0.0006 | Down |
| scaffold3.t282 | *TDA10*_Probable ATP-dependent kinase | 0.4376 | 0.0028 | Up |
| scaffold3.t385 | *HFD1*_Fatty aldehyde dehydrogenase | -0.3081 | 0.0000 | Down |
| scaffold3.t768 | *YDC1*_Alkaline ceramidase | -0.7868 | 0.0497 | Down |
| scaffold4.t136 | *CP51*_Lanosterol 14-alpha demethylase | -0.6280 | 0.0000 | Down |
| scaffold4.t172 | *AYR1*_NADPH-dependent 1-acyldihydroxyacetone phosphate reductase | -0.9776 | 0.0000 | Down |
| scaffold5.t437 | *PLSC*_Probable 1-acyl-sn-glycerol-3-phosphate acyltransferase | -0.3071 | 0.0010 | Down |
| scaffold5.t79 | *ERG5*_Cytochrome P450 61 | -0.4168 | 0.0000 | Down |
| scaffold6.t270 | *GEP4*_Phosphatidylglycerophosphatase | 0.3077 | 0.0126 | Up |
| scaffold6.t277 | *DPP1*_Diacylglycerol pyrophosphate phosphatase 1 | 0.3907 | 0.0138 | Up |
| scaffold6.t421 | *CHO2*_Phosphatidylethanolamine N-methyltransferase OX=294746 GN=CHO2 | -0.3438 | 0.0018 | Down |
| scaffold6.t83 | *ERG1*_Squalene monooxygenase | -0.8858 | 0.0000 | Down |
| scaffold7.t106 | *SUR2*_Sphingolipid C4-hydroxylase SUR2 | -0.4943 | 0.0001 | Down |
| scaffold7.t366 | *MSMO*_Methylsterol monooxygenase | -1.4331 | 0.0001 | Down |
| scaffold7.t388 | *CEGT*_Ceramide glucosyltransferase | 0.3061 | 0.0005 | Up |
| scaffold8.t78 | *ADH7*_NADP-dependent alcohol dehydrogenase 7 | 0.4746 | 0.0001 | Up |

**Supplementary Table S12:** Differential proteins under 5%-vs- 10% NaCl stress.

| Accession | Description | 5%-vs-10%-log2(fc) | Pvalue | Regulation |
| --- | --- | --- | --- | --- |
| scaffold7.t366 | *MSMO*_Methylsterol monooxygenase | -1.23192 | 0.001763 | Down |
| scaffold2.t903 | *ADH2*_Alcohol dehydrogenase 2 | -1.09935 | 3.33E-06 | Down |
| scaffold4.t136 | *CP51*_Lanosterol 14-alpha demethylase | -0.48922 | 2.13E-05 | Down |
| scaffold7.t106 | *SUR2*_Sphingolipid C4-hydroxylase | -0.47556 | 0.007592 | Down |
| scaffold5.t79 | *ERG5*_Cytochrome P450 | -0.4146 | 0.000304 | Down |
| scaffold6.t83 | *ERG1*_Squalene monooxygenase | -0.41202 | 0.000877 | Down |
| scaffold2.t347 | *MSMO*_Methylsterol monooxygenase | -0.40286 | 0.020006 | Down |
| scaffold6.t368 | *FABD*_Malonyl -acyl carrier | -0.35506 | 0.005752 | Down |
| scaffold3.t221 | *FAH12*_Oleate hydroxylase | -0.33696 | 0.004551 | Down |
| scaffold1.t611 | *SDR1*_Short-chain type dehydrogenase reductase | -0.32263 | 9.13E-05 | Down |
| scaffold1.t712 | *ACAC*_Acetyl- carboxylase | -0.29606 | 0.000123 | Down |
| scaffold2.t165 | *FAS2*_Fatty acid synthase subunit alpha | -0.29522 | 0.000315 | Down |
| scaffold3.t768 | *YDC1*_Alkaline ceramidase | -0.2853 | 0.047436 | Down |
| scaffold3.t569 | *TAZ1*_Lysophosphatidylcholine acyltransferase | 0.316954 | 0.000838 | Up |
| scaffold4.t172 | *AYR1*_NADPH-dependent 1-acyldihydroxyacetone phosphate reductase | 0.372886 | 0.000783 | Up |
| scaffold3.t282 | *TDA10*_Probable ATP-dependent kinase | 0.373129 | 0.001351 | Up |
| scaffold8.t78 | *ADH7*_NADP-dependent alcohol dehydrogenase 7 | 0.421245 | 6.09E-06 | Up |
| scaffold2.t925 | *THIKB*_3-ketoacyl- thiolase peroxisomal | 0.424093 | 0.000157 | Up |
| scaffold1.t695 | *EPT1*_Choline ethanolaminephosphotransferase 1 | 0.437216 | 0.024045 | Up |
| scaffold2.t541 | *LCF2*_Long-chain-fatty-acid-- ligase 2 | 0.511037 | 4.87E-06 | Up |
| scaffold2.t48 | *ADH3*_Alcohol dehydrogenase mitochondrial | 0.845954 | 0.019589 | Up |
| scaffold3.t157 | *ALDH5*_Aldehyde dehydrogenase | 1.080119 | 7.21E-05 | Up |

**Supplementary Table S13**: Enrichment of differential proteins in lipid metabolism under salt stress.

| Pathway | 0%-vs-5% | 0%-vs-10% | 5%-vs-10% |
| --- | --- | --- | --- |
| Steroid biosynthesis | 1 | 6 | 5 |
| Ether lipid metabolism | 2 | 2 | 2 |
| Fatty acid biosynthesis | 2 | 5 | 5 |
| Fatty acid degradation | 7 | 11 | 5 |
| Synthesis and degradation of ketone bodies | 0 | 1 | 0 |
| Glycerophospholipid metabolism | 6 | 7 | 3 |
| Glycerolipid metabolism | 4 | 7 | 3 |
| Biosynthesis of unsaturated fatty acids | 2 | 3 | 2 |
| alpha-Linolenic acid metabolism | 1 | 1 | 1 |
| Sphingolipid metabolism | 1 | 5 | 2 |
| Fatty acid elongation | 0 | 1 | 0 |

**Supplementary Table S14**: Association analysis of differential genes and differential proteins under 0%-vs- 5% NaCl stress.

| ProteinID | GeneID | Genes_log2(fc) | Genes_PValue | Proteins_log2(fc) | Proteins_Pvalue | Symbol | Description |
| --- | --- | --- | --- | --- | --- | --- | --- |
| scaffold4.t172 | scaffold4.g172 | -2.7602 | 0.0000 | -1.3505 | 0.0000 | *AYR1* | NADPH-dependent 1-acyldihydroxyacetone phosphate reductase |

**Supplementary Table S15**: Association analysis of differential genes and differential proteins under 0%-vs- 10% NaCl stress.

| ProteinID | GeneID | Genes_log2(fc) | Genes_PValue | Proteins_log2(fc) | Proteins_Pvalue | Symbol | Description |
| --- | --- | --- | --- | --- | --- | --- | --- |
| scaffold2.t903 | scaffold2.g903 | -1.4765 | 0.0000 | -1.4699 | 0.0000 | *ADH2* | Alcohol dehydrogenase 2 |
| scaffold4.t172 | scaffold4.g172 | -2.6886 | 0.0000 | -0.9776 | 0.0000 | *AYR1* | NADPH-dependent 1-acyldihydroxyacetone phosphate reductase |
| scaffold6.t83 | scaffold6.g83 | -2.3432 | 0.0000 | -0.8858 | 0.0000 | *ERG1* | Squalene monooxygenase |
| scaffold2.t165 | scaffold2.g165 | -2.1329 | 0.0000 | -0.7365 | 0.0000 | *FAS2* | fatty acid synthase alpha subunit |
| scaffold1.t712 | scaffold1.g712 | -2.2402 | 0.0000 | -0.6435 | 0.0000 | *ACC1* | Acetyl-CoA carboxylase |
| scaffold4.t136 | scaffold4.g136 | -1.1662 | 0.0000 | -0.6280 | 0.0000 | *ERG11* | Lanosterol 14-alpha demethylase |
| scaffold3.t221 | scaffold3.g221 | 1.2126 | 0.0000 | -0.6211 | 0.0001 | *FAH12* | delta(12)-fatty-acid desaturase |
| scaffold2.t48 | scaffold2.g48 | 1.2041 | 0.0000 | 1.1964 | 0.0082 | *ADH2* | Alcohol dehydrogenase 3 |
| scaffold3.t157 | scaffold3.g157 | 1.6809 | 0.0000 | 1.3866 | 0.0000 | *ALD5* | Potassium-activated aldehyde dehydrogenase |

**Supplementary Table S16**: Association analysis of differential genes and differential proteins under 5%-vs- 10% NaCl stress.

| ProteinID | GeneID | Genes_log2(fc) | Genes_PValue | Proteins_log2(fc) | Proteins_Pvalue | Symbol | Description |
| --- | --- | --- | --- | --- | --- | --- | --- |
| scaffold2.t903 | scaffold2.g903 | -1.4230 | 0.0000 | -1.0993 | 0.0000 | *ADH2* | Alcohol dehydrogenase 2 |
| scaffold3.t157 | scaffold3.g157 | 1.5202 | 0.0000 | 1.0801 | 0.0001 | *ALD5* | Potassium-activated aldehyde dehydrogenase |

**Supplementary Table S17**: Enrichment analysis of metabolic pathways of differential genes and differential proteins.

| Pathway | 0%-vs-5% | 0%-vs-10% | 5%-vs-10% |
| --- | --- | --- | --- |
| Fatty acid degradation | 0 | 3 | 2 |
| Fatty acid biosynthesis | 0 | 2 | 0 |
| Steroid biosynthesis | 0 | 2 | 0 |
| Ether lipid metabolism | 1 | 1 | 0 |
| Biosynthesis of unsaturated fatty acids | 0 | 1 | 0 |
| Glycerolipid metabolism | 0 | 1 | 1 |
| Glycerophospholipid metabolism | 1 | 1 | 0 |

**Supplementary Table S18**: Target genes and specific primers of RT-qPCR.

| Genes | Gene ID | Forward and reverse primer | PCR product size（bp） |
| --- | --- | --- | --- |
| *GUT1* | scaffold1.g955 | F：AATCACTCCCGAAGCCTATG  R：GGTTCTGGAAGCATTGGTAAC | 159 |
| *ADH7* | scaffold8.g78 | F：GTGGAAAGGAAAGTGAAGGG  R：CAGATTTGTGTTCACCGAGC | 166 |
| *GPP1* | scaffold3.g796 | F：ACTTTTTTGTGTGGAGCGTC  R：ACTTCTGGAACCTTGCCTTT | 163 |
| *Pichia kluyveri^[24]^* | Reference gene | F: AGTCTCGGGTTAGACGT  R： GCTTTTCATCTTTCCTTCACA | 169 |

**Supplementary Fig. S1:** Gene enrichment analysis of GXDK6.


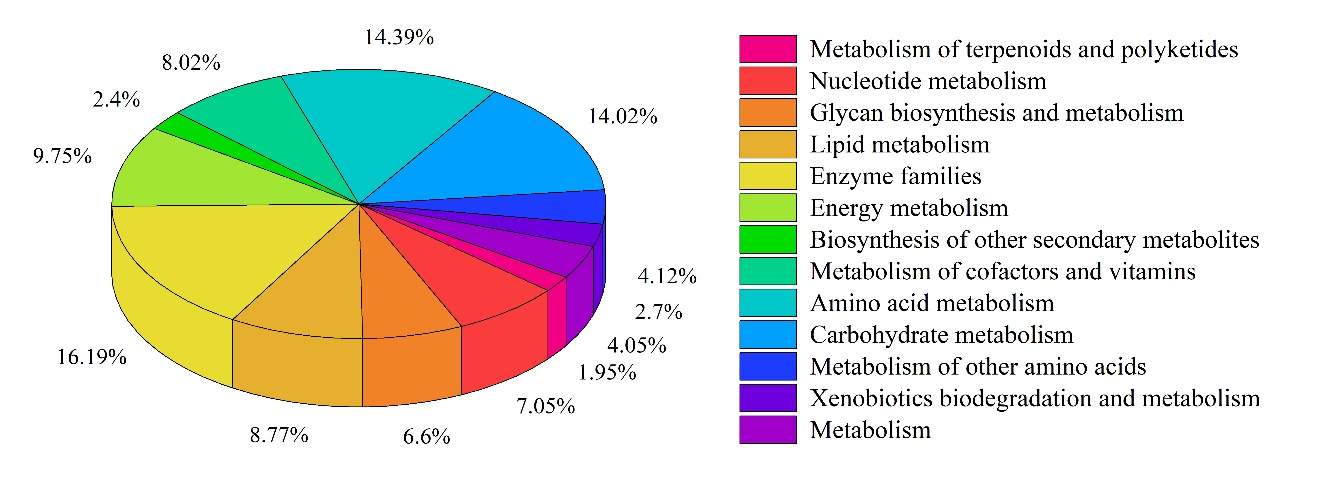


**Supplementary Fig. S2:** Differentially expressed genes in glycerolipid metabolism at 0%, 5%, and 10% NaCl.


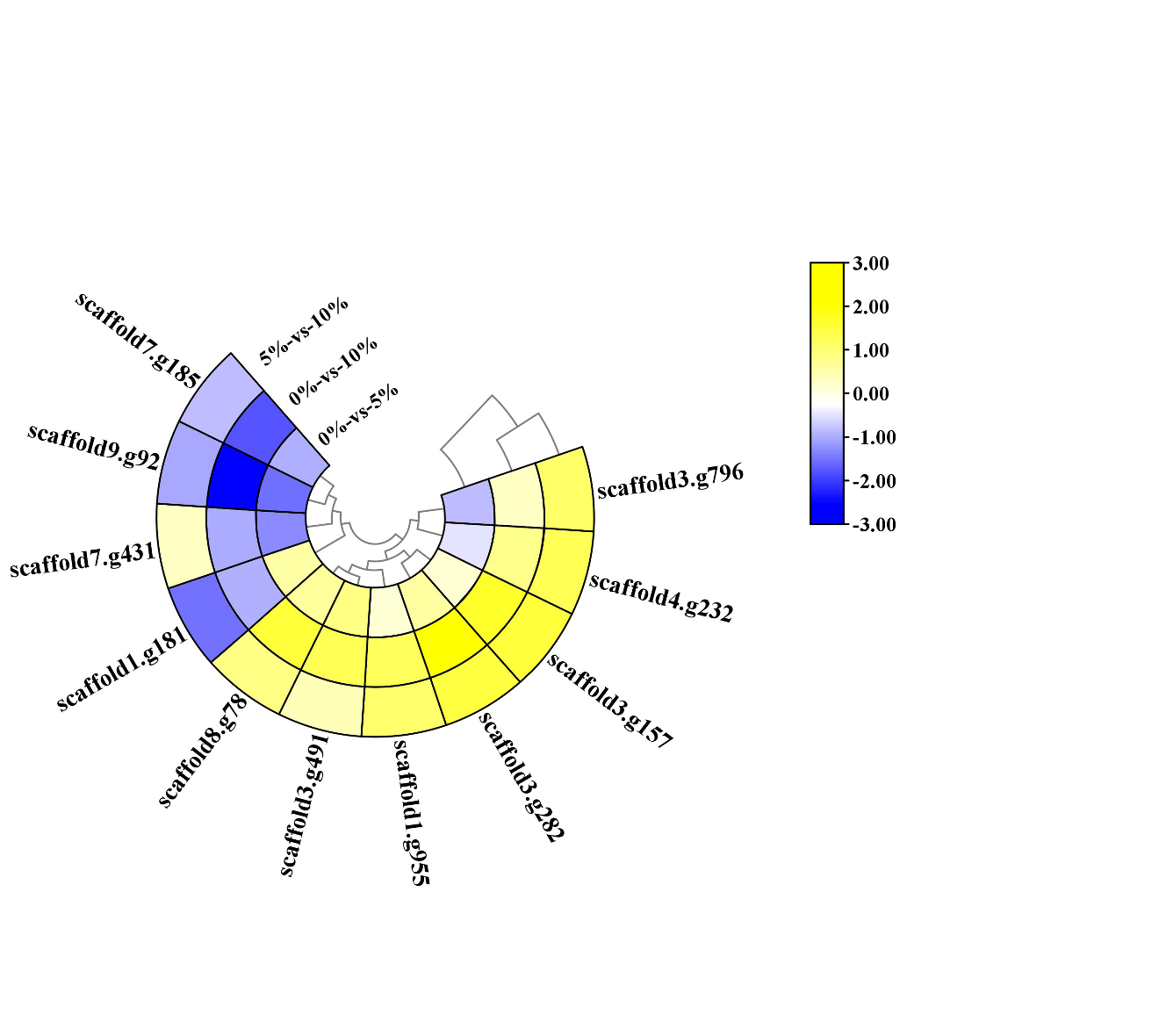


**Supplementary Fig. S3:** Effect of exogenous addition of glycerol on the growth of *M. guilliermondii.*
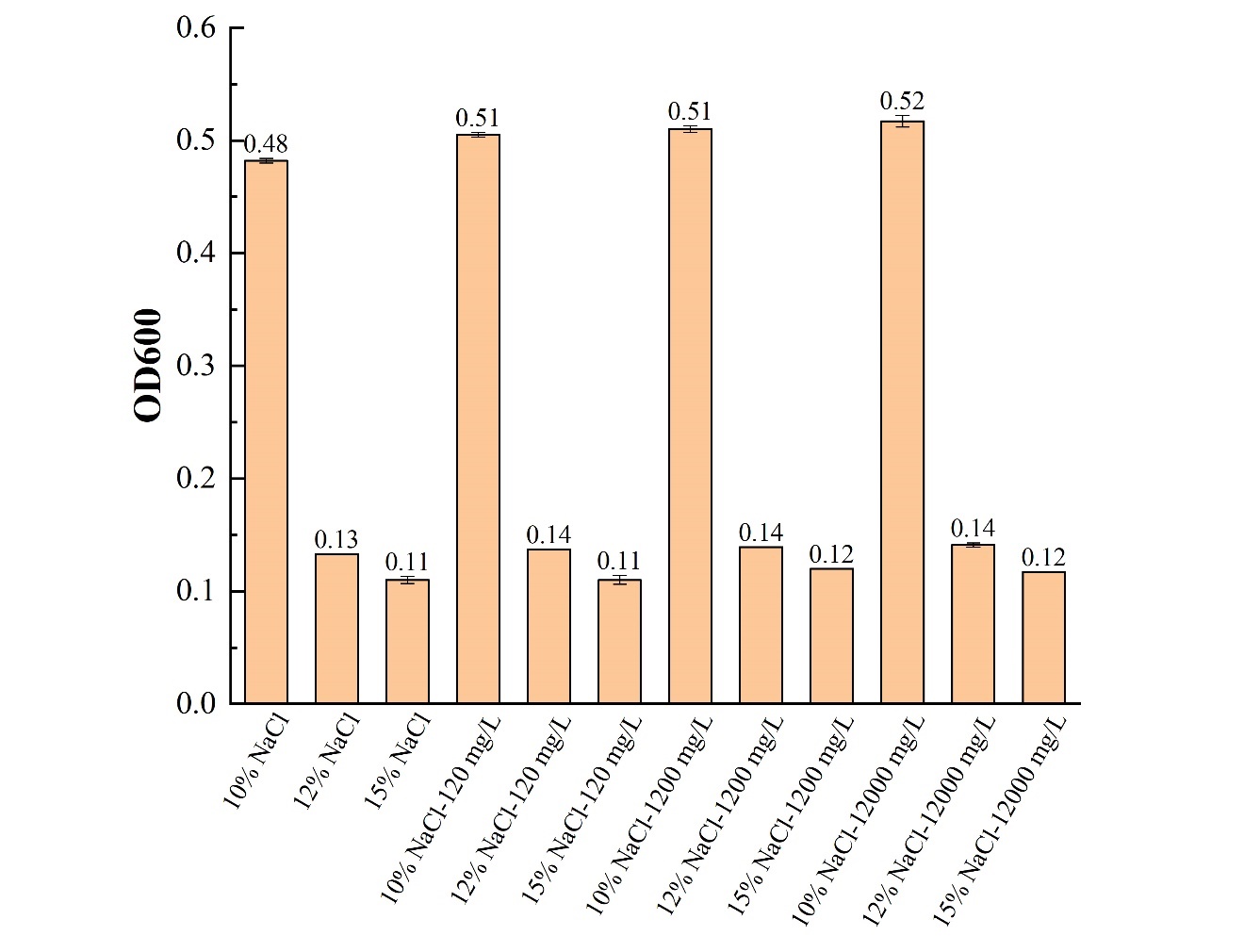


**Supplementary Fig. S4:** RT-qPCR verification results.


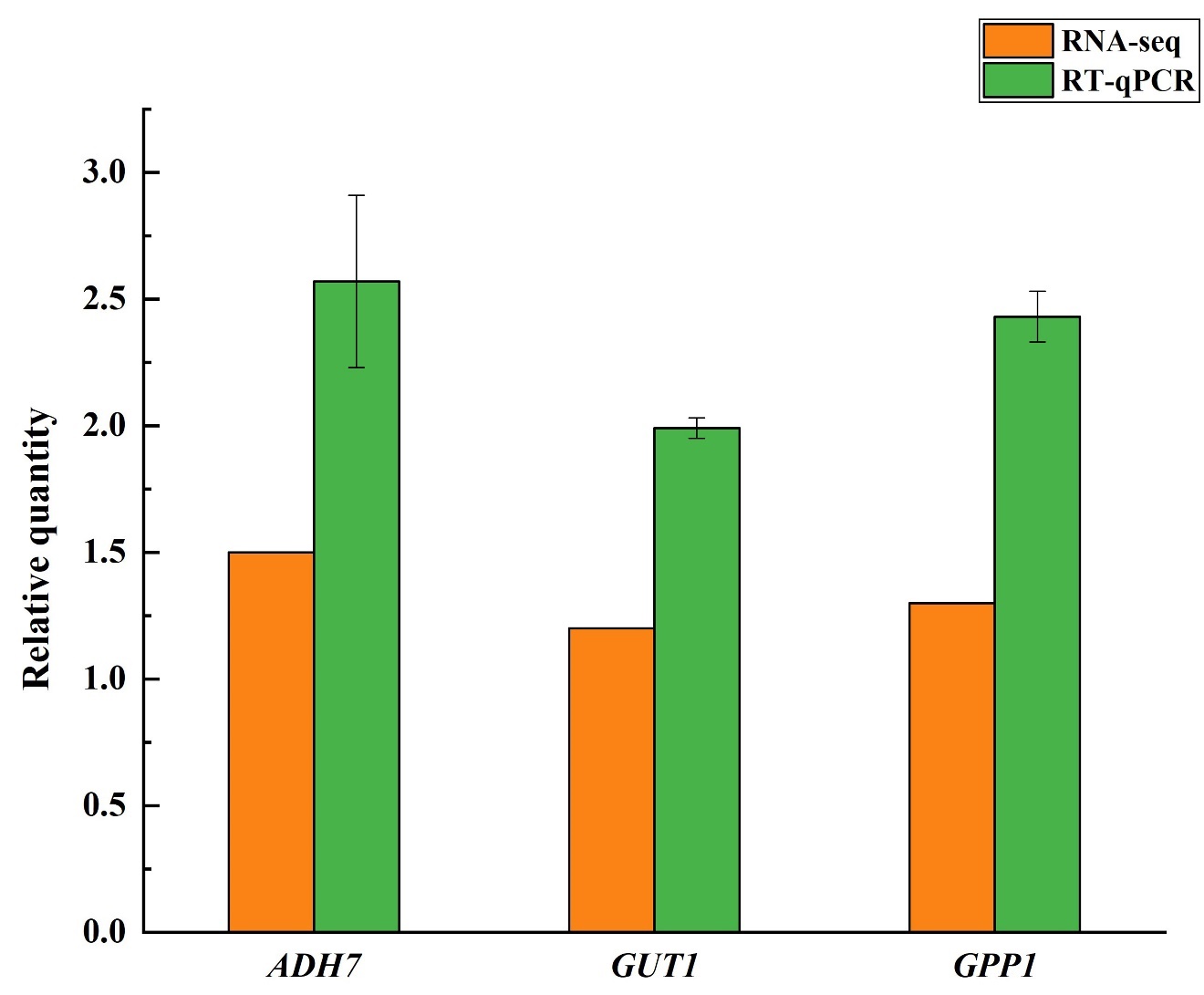


**Supplementary Fig. S5:** Drug resistance of GXDK6 to fluconazole under NaCl stress.





**Supplementary Fig. S6:** Drug resistance of GXDK6 to fluconazole under NaCl stress (colony). (A) 0%NaCl+0 μg/mL fluconazole; (B) 5%NaCl+0 μg/mL fluconazole; (C) 10%NaCl+0 μg/mL fluconazole; (D) 0%NaCl+64 μg/mL fluconazole; (E) 5%NaCl+64 μg/mL fluconazole; (F) 10%NaCl+64 μg/mL fluconazole.


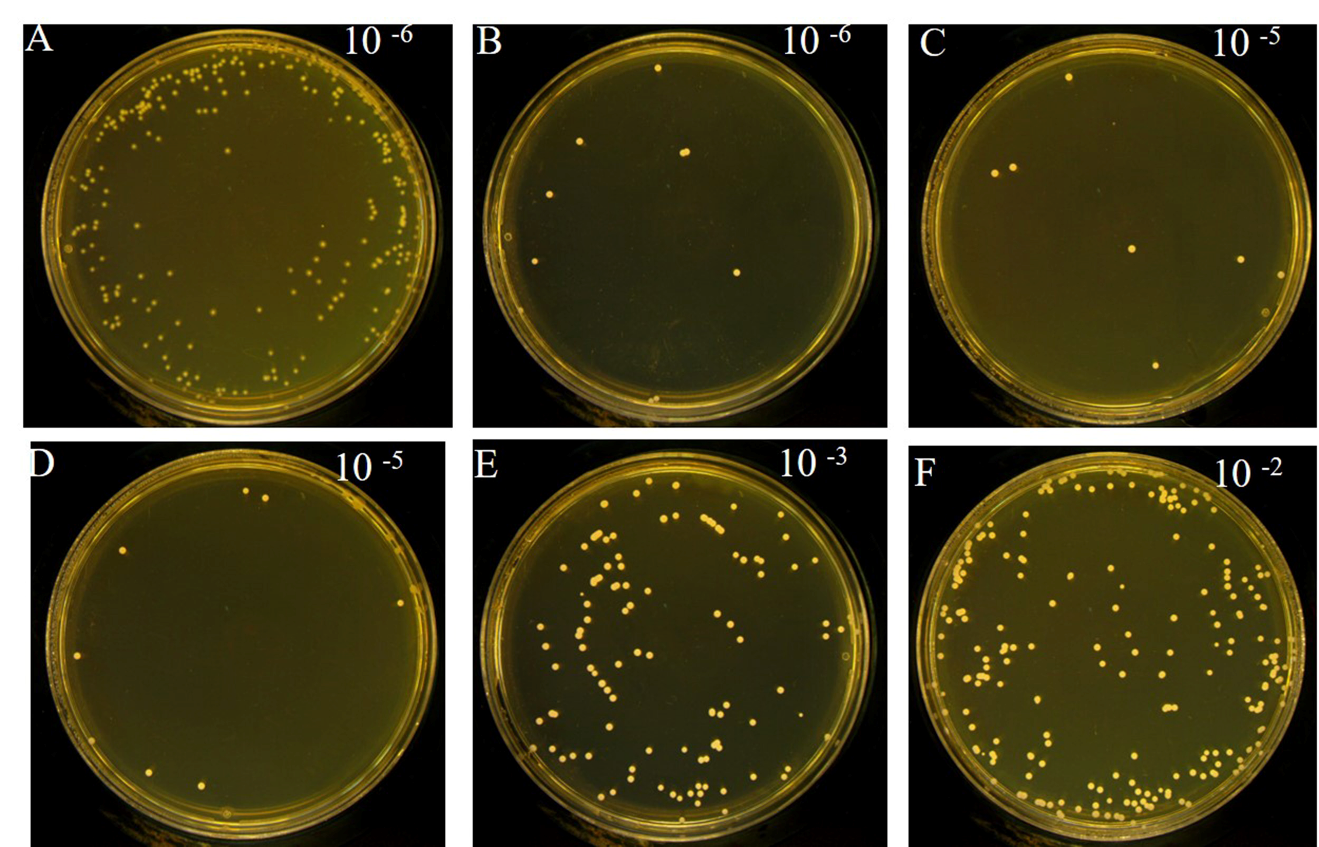

Supplement: Supplementary file 1 [file DataSheet1.docx]
